# Annexin A2/TLR2/MYD88 pathway induces arginase 1 expression in tumor-associated neutrophils

**DOI:** 10.1172/JCI153643

**Published:** 2022-11-15

**Authors:** Huajia Zhang, Xiaodong Zhu, Travis J. Friesen, Jeff W. Kwak, Tatyana Pisarenko, Surapat Mekvanich, Mark A. Velasco, Timothy W. Randolph, Julia Kargl, A. McGarry Houghton

**Affiliations:** 1 Clinical Research Division and; 2 Public Health Sciences Division, Fred Hutchinson Cancer Center, Seattle, Washington, USA.; 3 Otto Loewi Research Center, Division of Pharmacology, Medical University of Graz, Graz, Austria.; 4 Human Biology Division, Fred Hutchinson Cancer Center, Seattle, Washington, USA.; 5 Division of Pulmonary and Critical Care Medicine, University of Washington, Seattle, Washington, USA.

**Keywords:** Immunology, Oncology, Innate immunity, Lung cancer, Neutrophils

## Abstract

Myeloid lineage cells suppress T cell viability through arginine depletion via arginase 1 (ARG1). Despite numerous studies exploring the mechanisms by which ARG1 perturbs lymphocyte function, the cellular populations responsible for its generation and release remain poorly understood. Here, we showed that neutrophil lineage cells and not monocytes or macrophages expressed ARG1 in human non–small cell lung cancer (NSCLC). Importantly, we showed that approximately 40% of tumor-associated neutrophils (TANs) actively transcribed *ARG1* mRNA. To determine the mechanism by which *ARG1* mRNA is induced in TANs, we utilized FPLC followed by MS/MS to screen tumor-derived factors capable of inducing *ARG1* mRNA expression in neutrophils. These studies identified ANXA2 as the major driver of *ARG1* mRNA expression in TANs. Mechanistically, ANXA2 signaled through the TLR2/MYD88 axis in neutrophils to induce *ARG1* mRNA expression. The current study describes what we believe to be a novel mechanism by which *ARG1* mRNA expression is regulated in neutrophils in cancer and highlights the central role that neutrophil lineage cells play in the suppression of tumor-infiltrating lymphocytes.

## Introduction

In attempts to identify how solid tumors avoid immune-mediated destruction, several large-scale immune profiling studies have been performed on different human cancers utilizing various combinations of RNA sequencing, immunohistochemistry (IHC), whole-exome sequencing, flow cytometry, etc. (1–7). One common theme from such studies is the existence of an inflammatory or myeloid cell–infiltrated subset of cancers, in which there is a paucity of CD4^+^ and CD8^+^ lymphocytes and an absence of the favorable IFN-γ signature. Determining the mechanisms by which these tumors have achieved immune escape is of great importance to the immunotherapy field, as their identification may point to novel therapeutic targets with which to improve the relatively low response rates to immune checkpoint inhibitors observed in several cancer types.

Of the several plausible mechanisms to explain the above, evidence is greatest to support a role for myeloid lineage cells suppressing the ability of lymphocytes to proliferate or limiting their viability (8, 9). Such myeloid lineage cells are referred to as myeloid-derived suppressor cells (MDSCs), which can be subcategorized into those originating from monocytes (M-MDSCs) and those originating from neutrophil lineage cells (PMN-MDSCs) (9–12). Although a number of different M-MDSC– and PMN-MDSC–derived substances have been implicated in lymphocyte suppression in this context, reactive oxygen species (ROS) (13–15) and arginase 1 (ARG1) (16–18) have emerged as the 2 entities seemingly responsible for the majority of lymphocyte suppression in cancer. In fact, the data have supported such a potentially important role for ARG1 in this process that a phase I/II clinical trial is currently underway (ClinicalTrials.gov NCT02903914) testing the efficacy of an ARG1 inhibitor (INCB001158) with or without anti–PD-1 antibody (pembrolizumab) for metastatic solid tumors, including non–small cell lung cancer (NSCLC) (19).

Whereas the mechanisms by which ARG1 suppresses lymphocyte function have been well documented (e.g., L-arginine depletion and T cell receptor ζ chain downregulation; refs. 16, 20), the cellular sources of ARG1 protein and the regulation of ARG1 production in human disease remain poorly understood. Much of the confusion in the field likely stems from key differences in the ways in which ARG1 is transcribed, translated, stored, and released by human myeloid lineage cells as compared with their murine counterparts. Specific concerns include (a) *ARG1* gene expression serves as a prototypical marker of M2 macrophages in mice (21), though evidence that ARG1 is produced by human macrophages remains sparse; (b) debate regarding the cellular sources of ARG1 in humans is ongoing, as some groups believe its production is limited to neutrophil lineage cells (22, 23), while others have reported the existence of ARG1^+^ monocytes, at least in head and neck cancer (24); (c) of the numerous potential neutrophil populations in cancer patients and tumor-bearing mice (i.e., bone marrow [BM] neutrophils, splenic neutrophils, tumor-associated neutrophils [TANs], etc.), it remains unclear which subtypes produce *ARG1* transcripts and/or house ARG1 protein; and (d) human neutrophils typically make ARG1 protein in the BM and store it for later use in neutrophilic granules, though whether or not they produce ARG1 within disease environments such as the tumor microenvironment (TME) is unknown.

Clearly, a better understanding of ARG1 production and function mediated by myeloid lineage cells in NSCLC will be required to uncover the relevant mechanisms of immune escape, especially given that neutrophils are one of the most abundant immune cell types present within the lung TME (2).

Therefore, we undertook this study to definitively identify the cellular source(s) of ARG1 in human NSCLC, to determine whether TANs possessed the ability to produce ARG1 de novo, and to uncover the operative mechanisms involved in ARG1 production and function in this context. Surprisingly, we found that ARG1 activity is mostly derived from neutrophils in human NSCLC, that a sizeable neutrophil subset actively transcribes *ARG1* while located in the TME, and that *ARG1* transcripts are produced via a potentially novel annexin A2–mediated (ANXA2-mediated) pathway.

## Results

### Neutrophil lineage cells are the main source of ARG1 within the human lung TME.

**Figure 1:**
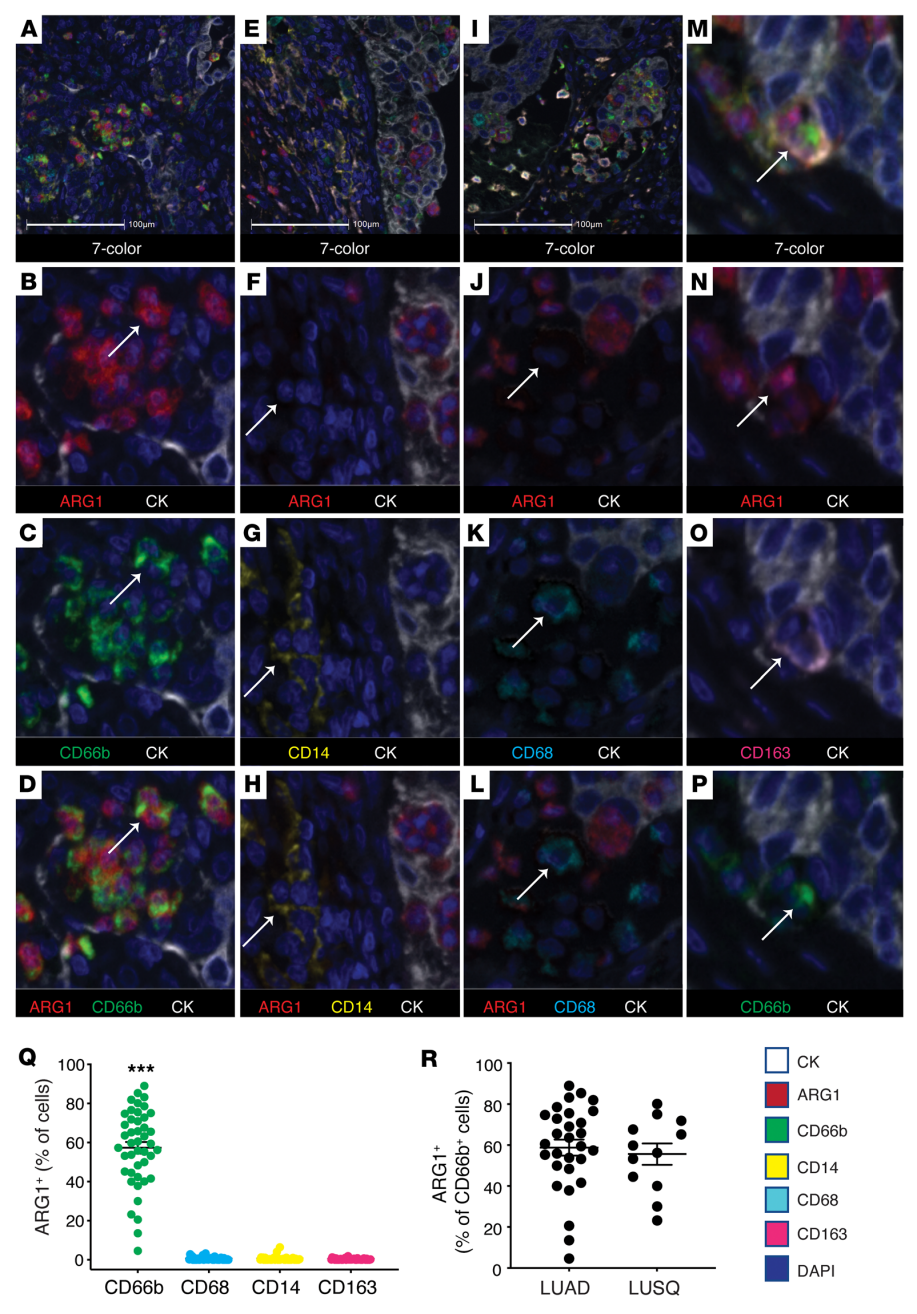
Arginase 1 is predominantly located within neutrophil lineage cells in human NSCLC. (**A**–**P**) Representative images from NSCLC cases (*n* = 44) stained for CD66b (green), CD68 (cyan), ARG1 (red), CD14 (yellow), CD163 (pink), AE1/AE3 (CK, white), and with DAPI (blue). Stained slides were imaged on the Vectra 3.0 platform and analyzed using HALO. (**A**–**D**) Depict ARG1 positivity within the CD66b^+^ population. (**E**–**H**) Depict ARG1 negativity within the CD14^+^ population. (**I**–**L**) Depict ARG1 negativity within the CD68^+^ population. (**M**–**P**) Depict a macrophage triple-positive for ARG1, CD163, and CD66b. Original magnification, ×10 (**A**, **E**, and **I**) and ×40 (all other panels). Scale bars: 100 μm. (**Q**) Percentage of ARG1^+^ cells in CD66b^+^ (green), CD68^+^ (cyan), CD14^+^ (yellow), and CD163^+^ (pink) cells quantified from FFPE human NSCLC slides, *n* = 44. ***P < 0.001 by 1-way ANOVA with Tukey’s post hoc test. (**R**) Percentage of ARG1^+^ cells in the CD66b^+^ population for LUAD (*n* = 32) and LUSQ (*n* = 12).

We generated a 7-color multiplexed immunohistochemical (M-IHC) panel on the Vectra 3.0 platform (PerkinElmer) in order to carefully document the distribution of ARG1 protein in the different immune cell subtypes within human NSCLC specimens (Figure 1). Panel workup and validation is provided in Supplemental Figure 1; supplemental material available online with this article; https://doi.org/10.1172/JCI153643DS1 Since there remains debate around the ability of human monocytes and/or macrophages to produce ARG1, we focused specifically on myeloid lineage cells and included the markers CD66b, CD14, CD68, and CD163 (Figure 1, A–P). Although CD68 and CD163 are not sufficient to distinguish M1 and M2 states by themselves, they do identify distinct and overlapping macrophage subtypes and allowed us to cover a broad range of macrophages for our purposes here. We observed that the majority of CD66b^+^ neutrophils, approximately 60%, stained positive for ARG1 (Figure 1Q). These results were not significantly different when comparing lung adenocarcinoma (LUAD) to lung squamous cell carcinoma (LUSQ) cases (Figure 1R). We identified negligible ARG1 staining in CD68^+^, CD163^+^, and CD14^+^ cells (Figure 1Q). The small numbers of these cells tabulated as ARG1^+^ in Figure 1Q likely represent borderline positive threshold issues, as manual review of the slides failed to produce a single CD68^+^, CD163^+^, or CD14^+^ cell with clear ARG1 positivity. The only exception to this were the few macrophages that were triple positive (CD68^+^CD66b^+^ARG1^+^ or CD163^+^CD66b^+^ARG1^+^). We speculate that the triple-positive cells that we observed represent macrophages that had phagocytized an ARG1^+^ neutrophil based on the morphology in M-IHC (Figure 1, M–P).

**Figure 2:**
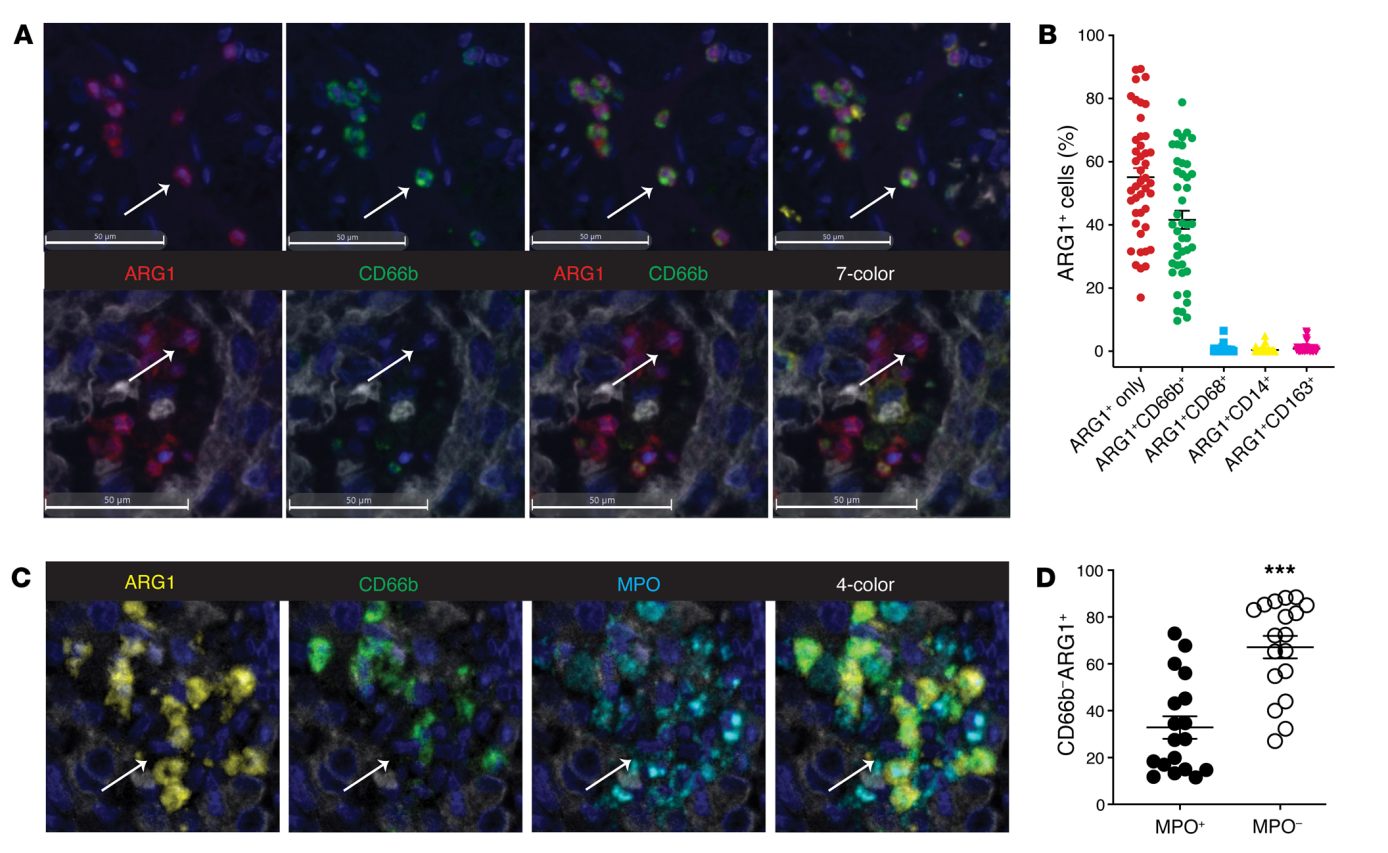
MPO marks a subset of CD66b^–^ARG1^+^ tumor-associated neutrophils. (**A**) Representative images from FFPE human NSCLC slides stained on the M-IHC platform for CK (white), ARG1 (red), CD14 (yellow), CD66b (green), CD68 (cyan), and CD163 (pink). The top panel depicts ARG1^+^ cells also staining positively for CD66b and the bottom panel depicts ARG1^+^ cells staining negatively for all other markers on the panel. Scale bars: 50 μm. (**B**) Percentage of each ARG1^+^ cell as a function of other panel markers as quantified from *n* = 44 NSCLC slides. Bars denote ± SEM. (**C**) Representative images from FFPE human NSCLC slides stained for ARG1 (yellow), CD66b (green), and MPO (cyan). (**D**) Tabulation of CD66b^–^ARG1^+^ cells as a function of MPO staining. *n* = 18. Bars denote ± SEM. ***P < 0.001 by 2-tailed Student’s *t* test. Original magnification, ×40 (all images).

It is noteworthy that of all ARG1^+^ cells identified here, approximately 45% of them stained positively for CD66b, but the other 55% did not stain positively for any of the markers on the panel (Figure 2, A and B). Manual review of these cells revealed that they were morphologically consistent with a neutrophil lineage cell that either did not express CD66b (below threshold limitation) or one in a necrotic or NETotic state (25). In order to address these concerns, we crafted an additional M-IHC panel including the marker myeloperoxidase (MPO) (based on the literature, at least a proportion of CD66b^–^MPO^+^ cells would represent neutrophil extracellular traps [NETs]) (26) and studied a subset of the NSCLC cohort (*n* = 12) to lend additional credence to our hypothesis. Although MPO can be expressed by myeloid lineage cells other than PMNs, we were not concerned about this overlap in staining since the data in Figure 1 conclusively demonstrated that CD14^+^, CD68^+^, and CD163^+^ cells did not stain positively for ARG1. Therefore, we did not include these markers on the MPO panel as it seemed quite unlikely that any MPO^+^ARG1^+^ cells would represent the monocyte/macrophage lineage.

As one would expect, the majority of CD66b^+^ neutrophils stained positively for MPO (Figure 2, C and D). However, closer examination of this panel also revealed heterogeneity within the ARG1^+^ population, with clear examples of CD66b^+^MPO^+^, CD66b^+^MPO^–^, and CD66b^–^MPO^+^ cells staining positively for ARG1. When we specifically examined the CD66b^–^ARG1^+^ population, we found that approximately 35% of these cells stained positively for MPO (Figure 2D). Alternatively, when we broadened the definition of a neutrophil to include any cell that expressed CD66b or MPO (or both), 75% of the ARG1^+^ population was accounted for (not shown). Taken together, these data suggest that the vast majority of the ARG1^+^ cellular population is confined to the neutrophil lineage, which would include traditional neutrophils, TANs, PMN-MDSCs, and NETs. Thus, the inability to account for the entirety of the ARG1^+^ population is likely the result of a lack of uniform marker expression by neutrophil lineage cells as opposed to the presence of an alternate ARG1-expressing immune cell type.

### TANs actively produce ARG1 transcripts in NSCLC.

**Figure 3:**
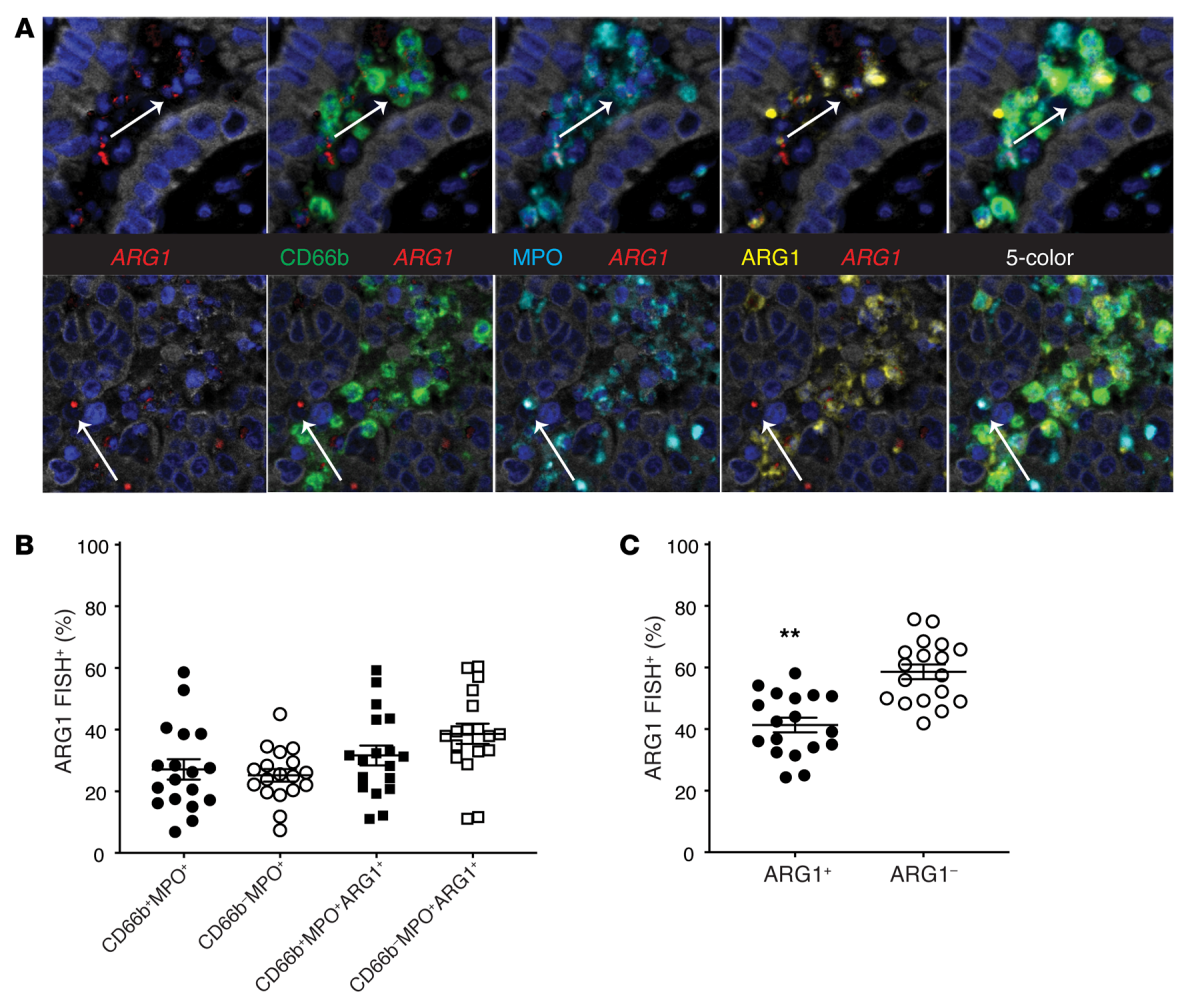
Tumor-associated neutrophils actively transcribe *ARG1*. (**A**) Representative images from FFPE NSCLC cases stained on combined M-IHC/FISH platform for ARG1-FISH (red), ARG1 IHC (yellow), CD66b (green), and MPO (cyan). Original magnification, ×40 (all images). (**B**) Tabulation of ARG1^–^FISH^+^ cells as a function of the other neutrophil markers on the panel. *n* = 18. Bars denote ± SEM. (**C**) Tabulation of ARG1^–^FISH^+^ neutrophils as a function of ARG1 protein staining by IHC. *n* = 18. Bars denote ± SEM. **P = 0.002, indicates comparison of Arg1^+^ value (41.36%) versus the null-hypothesis value (50%), by 2-tailed Student’s *t* test.

Current dogma would suggest that effectively all neutrophils produce ARG1 while in the BM and enter the circulation with ARG1 protein stored in granules (23). This paradigm is not specific for ARG1, as many neutrophil-derived enzymes are primarily produced during BM differentiation and stored in granules for later use (27). Based on the above findings, we suspected that some TANs had exhausted their cellular stores of ARG1 and questioned whether TANs could replenish their ARG1 via de novo *ARG1* gene transcription. It is believed that neutrophils can produce *ARG1* gene transcripts under certain inflammatory conditions (28), though whether this would occur within the TME and the mechanisms at play are unknown. These studies have been difficult to perform in humans because of the concern that gene expression studies involving neutrophils isolated from human cancers may not accurately reflect their in vivo behavior. These concerns reflect the facts that neutrophils do not tolerate cellular isolation protocols very well and display very short lifespans ex vivo, which is compounded by the inevitable processing time when human tumor resections are involved. Therefore, to determine whether the TME plays a role in ARG1 induction, we chose to craft an M-IHC panel combining ARG1 protein IHC and *ARG1* RNA fluorescent in situ hybridization (FISH) using RNAscope methodology (29). Panel workup and validation are provided in Supplemental Figure 2. This strategy allowed us to simultaneously detect both ARG1 protein and gene transcripts from TANs in vivo. The results, depicted in Figure 3, A and B, demonstrate that between 25% and 40% of TANs actively transcribe *ARG1*, once again depending on the exact marker definition of a neutrophil. Notably, TANs were statistically more likely to produce *ARG1* transcripts if they lacked ARG1 protein by IHC (Figure 3C). This process most likely represents a replenishment of ARG1 content following granule exocytosis, though it is possible that these represent immature neutrophils that have not completed the ARG1 production process that typically takes place within the BM. Regardless, we did not find evidence of a non-neutrophil source of *ARG1* transcripts. These studies demonstrate that TANs frequently produce *ARG1* mRNA, raising the possibility that TME factors are responsible for the induction of *ARG1* gene expression in TANs.

### TANs possess high levels of Arg1 activity in mice.

To follow up on the finding that TANs actively transcribe *ARG1* mRNA in human NSCLC, we chose to identify the exact mechanism by which this event occurs. Since ARG1 production and activity are mainly limited to neutrophil lineage cells in humans, we limited further investigation of ARG1 expression and function to neutrophils. We chose to conduct these experiments in mice since mouse tumor models allow for greater experimental control and provide the option of using genetically manipulated models to optimally determine operative mechanisms. Lastly, unlike human neutrophils, murine PMNs typically produce *Arg1* transcripts in response to external stimuli (akin to the above finding of ARG1-FISH^+^ TANs in human NSCLC), and do not routinely produce ARG1 while in the BM nor store it in granules for later use. As a result of these features, mouse neutrophils tend to display pronounced induction of *Arg1* mRNA under the right experimental conditions. Therefore, mouse neutrophils provide a robust readout for ARG1 expression that is ideal when screening candidate TME factors for their ability to induce *Arg1* transcripts.

We utilized 2 mouse models of lung cancer to investigate the ARG1 profile in neutrophil lineage cells: the *Lkb1^fl/fl^*; *Pten^fl/fl^* (PL) model of LUSQ and the Lewis lung carcinoma (LLC) allograft model of LUAD. These models were chosen for their close resemblance to human disease and their relatively high degree of PMN infiltration (30, 31). Initially, we utilized flow cytometry to determine which neutrophil subsets harbored ARG1 protein in the context of lung cancer. Because neutrophils in cancer patients and tumor-bearing mice display a high level of heterogeneity and plasticity (8), we examined all of the major neutrophil populations: BM PMNs (BM-PMNs), splenic PMNs (SP-PMNs), TANs, and peripheral blood PMNs. In the setting of cancer, these peripheral blood PMNs can be subdivided using Ficoll separation into high-density neutrophils (HDNs, neutrophils) and low-density neutrophils (LDNs, also known as neutrophilic MDSCs or PMN-MDSCs) (32).

**Figure 4:**
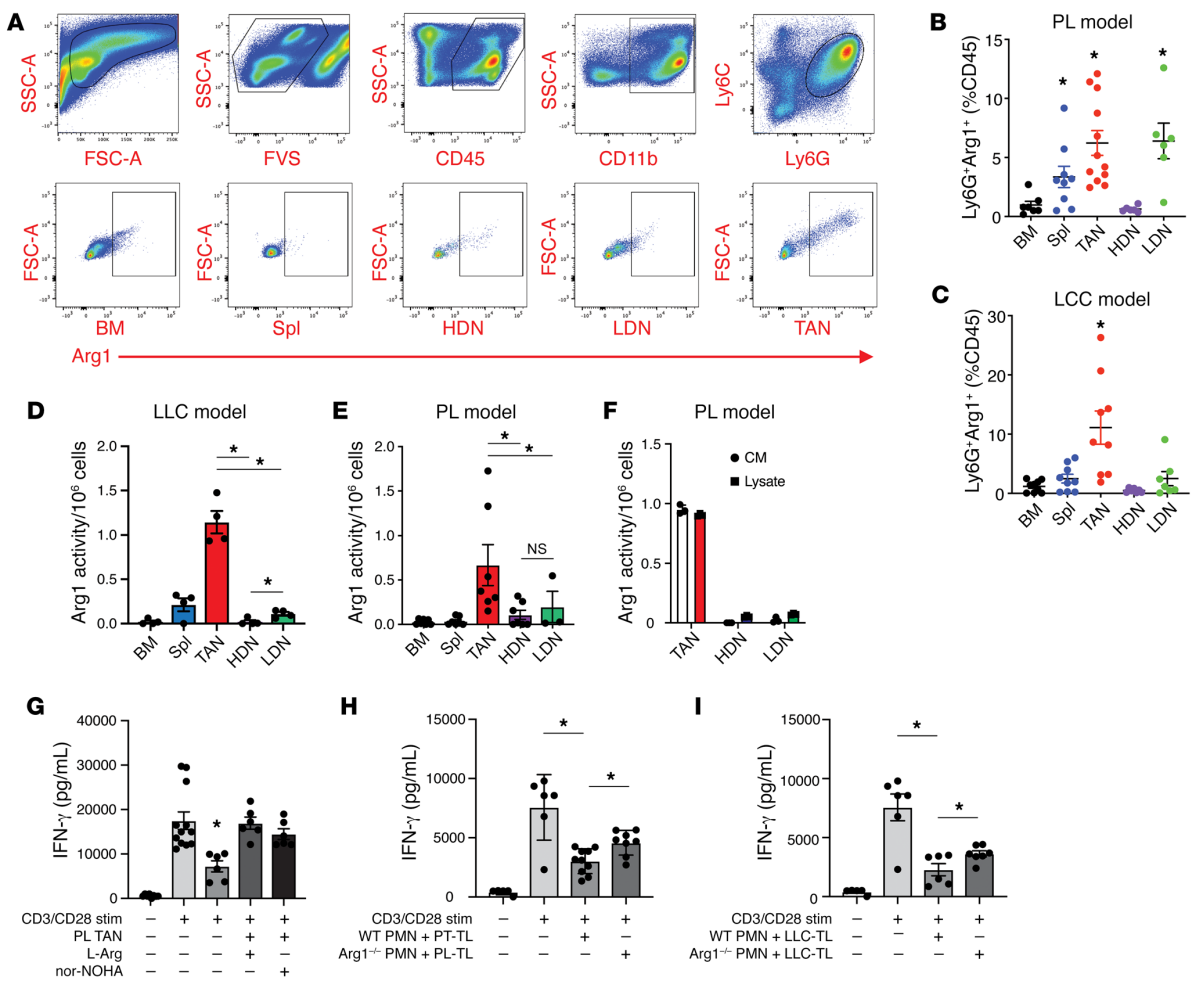
Tumor-associated neutrophils possess high levels of ARG1 activity. (**A**) Gating strategy utilized to identify tumor-associated neutrophils (TANs) and ARG1^+^ cells from tumor single-cell suspensions. Percentage of ARG1^+^ cells in PMNs from the BM, spleen (Spl), tumor (TAN), HDN, and LDN subsets in (**B**) PL-tumor-bearing (BM, *n* = 7; SP, *n* = 9; tumor, *n* = 12; HDN, *n* = 6; LDN, *n* = 6) and (**C**) LLC-tumor-bearing (BM, *n* = 9; Spl, *n* = 9; TAN, *n* = 9; HDN, *n* = 7; LDN, *n* = 7) mice. *P < 0.05 compared with BM control. ARG1 activity assay for the lysates of column-purified PMNs (BM, Spl, TAN, HDN, LDN) from (**D**) LLC-tumor-bearing (*n* = 4 each group) and (**E**) PL-tumor-bearing (BM, Spl, TAN, HDN, *n* = 7; LDN, *n* = 3) mice and from (**F**) the conditioned medium (CM) from TANs, HDNs, and LDNs isolated from PL-tumor-bearing mice and incubated overnight. *n* = 4 per group. Data expressed as ARG1 activity per 1 × 10^6^ PMNs from each neutrophil subset. (**G**) IFN-γ activity assay for column-purified splenic CD8^+^ T cells (*n* = 12), with anti-CD3/anti-CD28 activation (1 μg/mL) (*n* = 12), coculture with TANs (ratio 1:1, *n* = 6), supplemented with 75 μM L-arginine (*n* = 6) and with 10 μM ARG1 inhibitor (nor-NOHA, *n* = 6). IFN-γ assay as above with anti-CD3/anti-CD28 stimulation, cocultured with (**H**) PL-TLs activated WT HDNs or HDNs isolated from *Ly6G-Cre/Arg1^fl/fl^* mice (*n* = 6–8 per group) or (**I**) LLC-TLs activated WT HDNs or HDNs isolated from *Ly6G-Cre/Arg1^fl/fl^* mice (*n* = 6 per group). *P < 0.05 by 1-way ANOVA with Tukey’s post hoc test.

**Figure 5:**
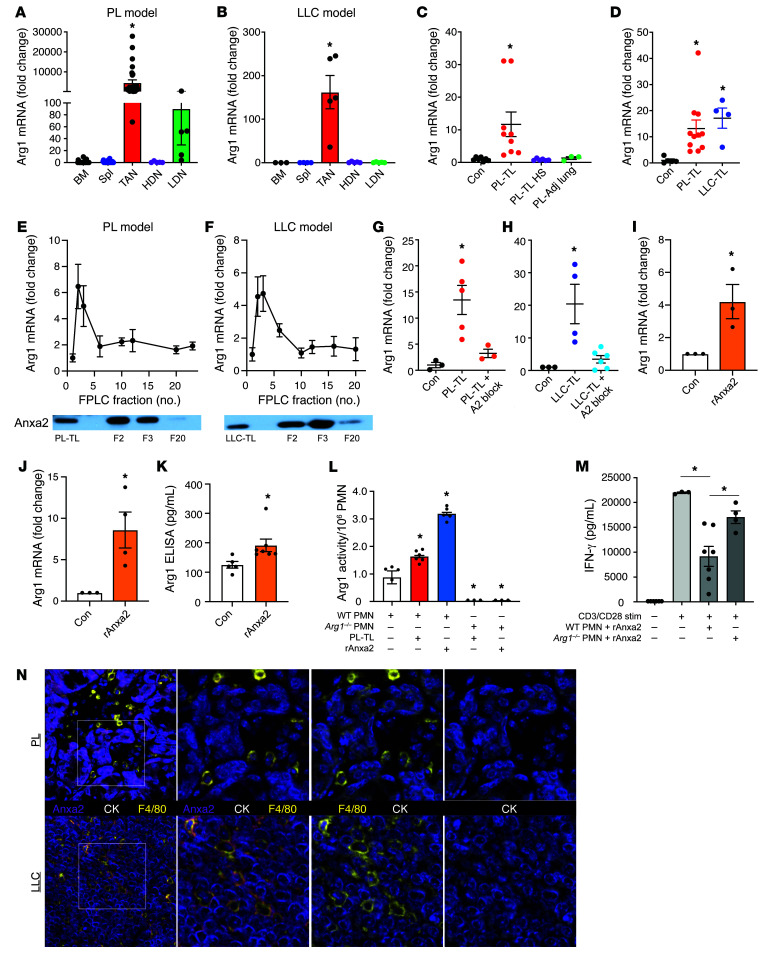
Tumor-derived ANXA2 drives *ARG1* gene expression in tumor-associated neutrophils. *Arg1* mRNA expression in PMNs from the BM, spleen (Spl), tumor (TANs), HDNs, and LDNs from (**A**) PL-tumor-bearing (BM, *n* = 25; Spl, *n* = 28; TAN, *n* = 28; HDN, *n* = 5; LDN, *n* = 5) and (**B**) LLC-tumor-bearing mice (BM, *n* = 3; Spl, *n* = 4; TAN, HDN, LDN, *n* = 5). (**C**) C57BL/6 BM PMNs treated with PBS (*n* = 7), PL tumor lysate (PL-TL, 100 μg/mL, *n* = 9), heat-shocked PL-TL (100 μg/mL, *n* = 6) or adjacent lung lysate (100 μg/mL, *n* = 3) for 16 hours. Data expressed as fold change in *Arg1* mRNA expression compared with PBS control. (**D**) C57BL/6 HDNs treated with PBS (*n* = 5), PL-TL (100 μg/mL, *n* = 11), and LLC-TL (100 μg/mL, *n* = 4) for 16 hours. Data expressed as fold change in *Arg1* mRNA expression compared with PBS control. C57BL/6 BM PMNs treated with equal volume (100 μL) of FPLC fractions from (**E**) PL-TL or (**F**) LLC-TL for 16 hours. Data expressed as fold change in *Arg1* mRNA expression compared with PBS control (*n* = 3). (**E** and **F**) Representative Western blot for ANXA2 in FPLC fractions 2, 3, and 20 from PL-TL and LLC-TL, respectively. C57BL/6 HDNs treated with (**G**) PL-TL (100 μg/mL) and (**H**) LLC-TL (100 μg/mL) in the presence of control peptide (LGKLSK, 10 μM) or ANXA2-blocking peptide (LCKLSK, 10 μM) for 16 hours. Data expressed as fold change in *ARG1* gene expression compared with PBS control. (**I**) BM PMNs (*n* = 3) and (**J**) HDNs (*n* = 4) isolated from C57BL/6 mice treated with recombinant mouse ANXA2 (rANXA2; 1 μg/mL) for 16 hours. Data expressed as fold change in *Arg1* mRNA expression compared with PBS control. (**K**) CM from HDNs isolated and treated as in **J** and analyzed by ARG1 ELISA (control, *n* = 5; rANXA2, *n* = 7). Data expressed as ARG1 protein in pg/mL. (**L**) ARG1 activity assay present in the CM for WT and *Arg1^–/–^* PMNs incubated with vehicle, PL-TL, or rANXA2. *n* = 5 per group. *P < 0.05 compared with control. (**M**) IFN-γ activity assay for column-purified splenic CD8^+^ T cells (*n* = 5), with anti-CD3/anti-CD28 activation (1 μg/mL) (*n* = 5), and coculture with WT (*n* = 7) or *Arg1^–/–^* (*n* = 4) PMNs at a 1:1 ratio. (**N**) Representative images for FFPE slides from PL and LLC tumors (*n* = 4 each) stained for ANXA2 (purple), F4/80 (yellow), CK (white), and with DAPI (blue). *P < 0.05 by 1-way ANOVA with Tukey’s post hoc test (**A**–**D**, **G**, **H**, **L**, and **M**) or 2-tailed Student’s *t* test (**I**).

Flow cytometric analysis showed minimal ARG1 intracellular protein content in naive BM-PMNs and HDNs (Figure 4, A–C). The intracellular level of ARG1 was significantly higher in SP-PMNs in both PL- and LLC-tumor-bearing mice. This observation is consistent with a previous finding that some SP-PMNs from tumor-bearing mice exhibit T cell suppressive properties (32). LDNs from PL mice showed a significantly higher ARG1 level compared with BM-PMNs and HDNs, while LDNs from LLC mice trended toward an increased level of ARG1 that did not reach statistical significance. This is consistent with previously published data showing that LDNs manifest myelosuppressive properties. Not surprisingly, TANs from both tumor models possessed the largest population of ARG1^+^ cells (Figure 4, A–C). Since flow cytometry qualitatively identifies cellular ARG1 positivity and not ARG1 content, per se, we analyzed the different neutrophil populations in an ARG1 activity assay, which measures functional ARG1 protein content. Neutrophils from the BM, spleen, tumor, and blood (HDNs and LDNs) were column purified to greater than 95% purity (data not shown). ARG1 activity in TAN lysates and conditioned medium (CM; secreted from PMNs during 6-hour incubation ex vivo) was several-fold higher than in any of the other subsets (e.g., HDNs) in PL mice (Figure 4, D and E). We observed similar findings in the TAN lysates in the LLC model (Figure 4F). Notably, the ARG1 expressed by TANs was functionally immunosuppressive, as evidenced by reduced IFN-γ secretion by stimulated splenic CD8^+^ T cells ex vivo (Figure 4G). This response was prevented by the addition of L-arginine (75 μM) or the ARG1 inhibitor N^ω^-hydroxy-nor-arginine (nor-NOHA, 10 μM). Similarly, HDNs activated by PL or LLC tumor lysates (TLs) also reduced IFN-γ secretion by CD8^+^ cells (Figure 4, H and I), suggesting that a TME factor is responsible for neutrophilic ARG1 expression in this context. To carefully control for the role of ARG1 in this process, we generated *Ly6G-Cre-Arg1^fl/fl^* mice (by crossbreeding *Ly6G-Cre* and *Arg1^fl/fl^* mice). *Ly6G-Cre-Arg1^fl/fl^* neutrophils lacked arginase activity (Figure 5K) but displayed normal function with respect to chemotaxis, phagocytosis, and peripheral blood content (Supplemental Figure 3). Using HDNs from these mice in the PMN–CD8^+^ cell coculture assays showed that ARG1 is responsible for approximately 50% of the suppressive activity of neutrophils (Figure 4, H and I).

### Tumor-derived ANXA2 drives Arg1 gene expression in neutrophils.

Based on the above data and what is known regarding ARG1 expression in mouse neutrophils, we suspected that *Arg1* mRNA expression would be highest in TANs and more or less negligible in the other populations. Accordingly, qPCR analysis showed that the *Arg1* mRNA level was over 100-fold higher in TANs as compared with the other neutrophil subsets in both PL- and LLC-tumor-bearing mice (Figure 5, A and B). This observation confirmed our suspicion that certain factor(s) within the TME dictated *Arg1* mRNA levels in PMNs upon their recruitment to the TME. To test this hypothesis, we isolated naive BM-PMNs and HDNs from tumor-free mice and cultured them with TLs generated from PL tumors ex vivo (PL-TLs). We found that PL-TLs induced a 10-fold increase in *Arg1* mRNA expression (Figure 5C). To determine whether the putative factor(s) is protein based, we treated the TLs with heat shock (95°C, 5 minutes). Heat-shocked TLs failed to induce *Arg1* mRNA expression, suggesting that the operative factor was indeed of protein origin. Furthermore, lysates generated from adjacent but noncancerous lung tissue from PL mice did not induce *Arg1* transcript production, suggesting that the operative factor was tumor specific (Figure 5C). Both PL-TLs and LLC-TLs were able to induce *Arg1* mRNA in naive HDNs, demonstrating that this process is not tumor model specific (Figure 5D).

Since multiple studies have reported that various cytokines — prostaglandin E_2_, IL-1β, IL-4, IL-13, IL-10, and others (33–36) — can induce *Arg1* mRNA expression, we analyzed PL-TLs and LLC-TLs using a cytokine profiling array. However, none of the previously reported ARG1-inducing cytokines were observed on the array or shown to be differentially presented in the tumor and adjacent lung (Supplemental Figure 4). Therefore, in order to identify the operative factor, we performed fast protein liquid chromatography (FPLC) on PL-TLs and LLC-TLs followed by mass spectrometry (MS). Briefly, lysate samples (1 mg/mL) were resolved by FPLC column separation based on molecular weight and 23 flow-through fractions (F1–F23) were collected from each lysate. An equal volume (100 μL) of F1–F23 was used to treat BM-PMNs from tumor-free B6 mice followed by qPCR for *Arg1* gene expression. Notably, F2 and F3 from both TLs (PL and LLC) demonstrated the ability to induce *Arg1* mRNA in naive PMNs (Figure 5, E and F), suggesting that the ARG1-inducing molecule(s) was concentrated in F2 and F3. To identify the operative molecule(s), we performed MS analysis on F2 and F3 from PL-TLs and LLC-TLs. More than 400 proteins were identified in both fractions using a 1% false-discovery rate at the peptide level (37). Many of the identified proteins were cellular structural and organelle proteins associated with mitochondria, ribosomes, and the endoplasmic reticulum. These proteins were excluded from the list of potential candidates. We screened the remaining MS data for proteins found in each of the 4 fractions and generated a list of candidates summarized in Table 1. The 4 common proteins present in both F2 and F3 are highlighted in bold. These 4 proteins are MPO, Ras-related protein Rab-11B (RAB11B), ANXA2, and integrin β1 (ITGB1). Of the 4 proteins, ANXA2 was the protein most likely responsible for ARG1 induction, as it has been reported to be a ligand for Toll-like receptor 2 (TLR2) and TLR4 (38, 39) and because TLR activation has been associated with the induction of *Arg1* mRNA expression (40). Western blotting confirmed that ANXA2 was highly enriched in F2 and F3 from both PL-TLs and LLC-TLs as opposed to fractions that did not induce *Arg1* transcript production (Figure 5, E and F, inset).

To test whether ANXA2 in PL and LLC tumors played a role in the induction of *Arg1* gene expression in neutrophils, we stimulated neutrophils with PL-TLs and LLC-TLs in the presence of an ANXA2 inhibitory peptide (LCKLSK, 10 μM) and control peptide (LGKLSK, 10 μM) (41). The ANXA2 inhibitory peptide blunted the ARG1 induction response triggered by TLs, while the control peptide did not (Figure 5, G and H). Naive BM-PMNs and HDNs were treated with recombinant mouse ANXA2 (1 μg/mL), which significantly induced *Arg1* mRNA expression (Figure 5, I and J). Since active ARG1 protein must be secreted from the cell of origin to exert its effect on extracellular arginine, we assessed ARG1 content within the cell (lysate) and secreted from the cell (CM) in response to ANXA2 stimulation using both ELISA (Figure 5K) and arginase activity assay (Figure 5L). The results confirmed that active ARG1 protein was produced and secreted in response to ANXA2 stimulation. ANXA2-stimulated neutrophils displayed lymphocyte inhibitory properties (as measured by reduction in IFN-γ release) that is mostly dependent on ARG1 (Figure 5M), demonstrating the relevance of ANXA2 stimulation on neutrophil function. Lastly, we crafted an abbreviated M-IHC panel to identify the cellular source of ANXA2 in the mouse models employed here. The results showed that ANXA2 is predominantly expressed by tumor cells (cytokeratin^+^, CK^+^) in both the PL and LLC models, with very little staining evident in macrophages (F4/80^+^) (Figure 5N).

### ANXA2 induction of Arg1 mRNA expression requires TLR2/MYD88 signaling.

**Figure 6:**
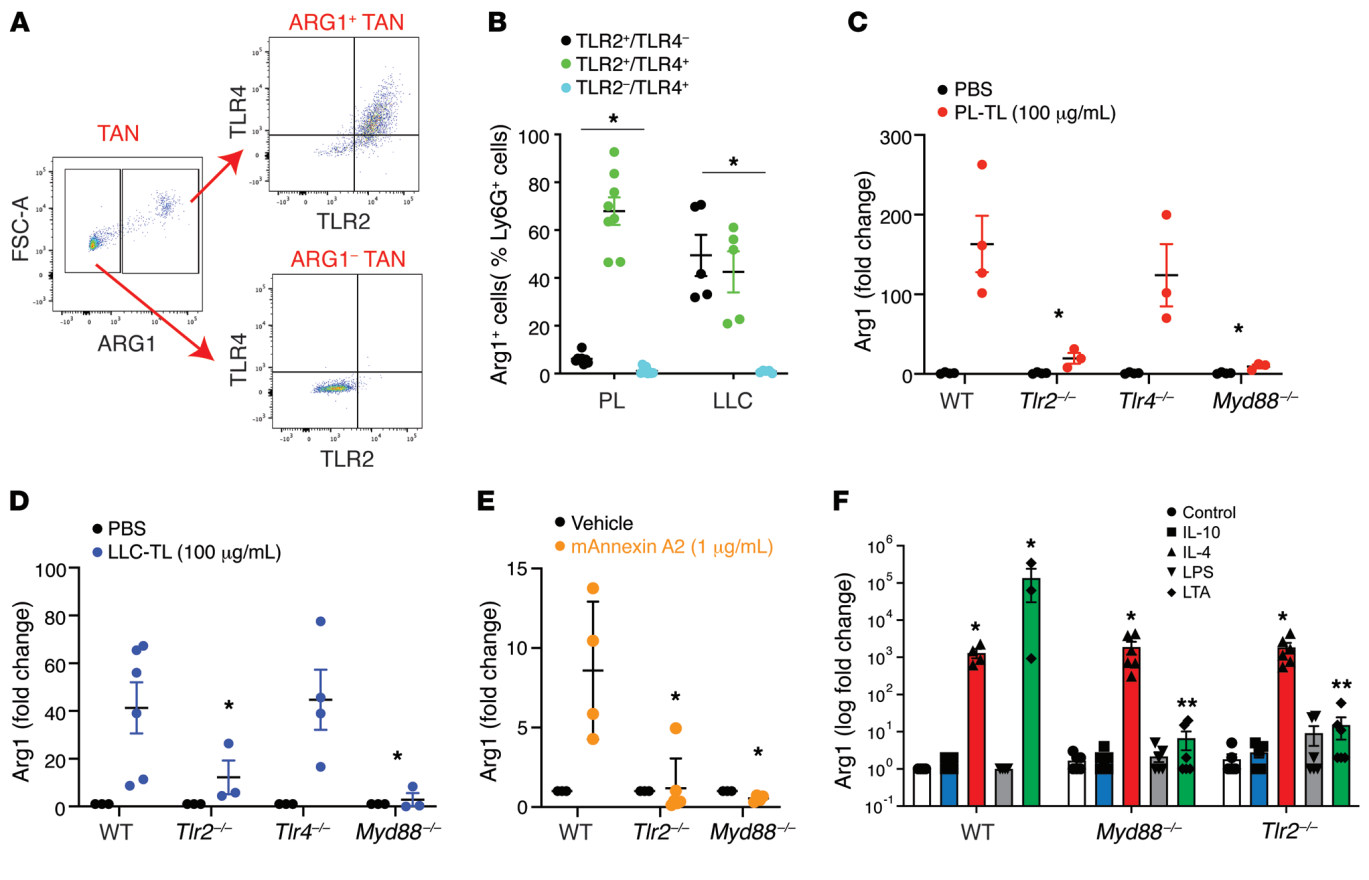
ANXA2 induction of *Arg1* mRNA expression requires TLR2/MYD88 signaling. (**A**) Representative dot plots depicting TLR2 and TLR4 expression in ARG1^+^ and ARG1^–^ TANs from PL mice. (**B**) Tabulation of percentage of TLR2^+^TLR4^–^, TLR2^+^TLR4^+^, and TLR2^–^TLR4^+^ cells in the ARG1^+^ TANs from PL-tumor-bearing (*n* = 8) and LLC-tumor-bearing (*n* = 5) mice. (**C**) Peripheral blood HDNs from tumor-free WT, *Tlr2^–/–^*, *Tlr4^–/–^*, and *Myd88^–/–^* mice were treated with PL-TL (*n* = 3–6) or (**D**) LLC-TL (*n* = 4–8) (100 μg/mL) for 16 hours. Data expressed as *Arg1* mRNA expression fold change in TL-treated HDNs compared with PBS control. (**E**) Peripheral blood HDNs from tumor-free WT, *Tlr2^–/–^*, and *Myd88^–/–^* mice were treated with recombinant mouse ANXA2 (1 μg/mL) for 16 hours. Data expressed as *Arg1* mRNA expression fold change in ANXA2-treated HDNs compared with PBS control (*n* = 4–6). (**F**) Peripheral blood HDNs from tumor-free WT, *Tlr2^–/–^*, and *Myd88^–/–^* mice were treated with IL-10 (10 U/mL), IL-4 (10 U/mL), LPS (0.1 μg/mL), LTA (20 μg/mL), or vehicle control for 16 hours. Data expressed as *Arg1* mRNA expression fold change versus PBS control (*n* = 4–6 per group). *P < 0.05 by 1-way ANOVA with Tukey’s post hoc test.

ANXA2 is a 36 kDa Ca^2+^-dependent phospholipid-binding protein that functions as a monomer or heterotetramer in combination with S100A10 (42). ANXA2 monomer binds to both TLR2 and TRL4 as a danger-associated molecular pattern (DAMP), with downstream signaling through adaptor protein myeloid differentiation 88 (MYD88) (38, 39). In our tumor models, both TLR2 and TRL4 were expressed on ARG1^+^ TANs but absent on ARG1^–^ TANs (Figure 6, A and B).

To test the hypotheses that ANXA2 was signaling via TLR2/MYD88 or TLR4/MYD88, we obtained *Tlr2^–/–^*, *Tlr4^–/–^*, and *Myd88^–/–^* mice from The Jackson Laboratory for the purposes of studying the impact of these gene products on neutrophilic production of ARG1. Neutrophils (HDNs) isolated from WT, *Tlr2^–/–^*, *Tlr4^–/–^*, and *Myd88^–/–^* mice were exposed to PL-TLs and LLC-TLs ex vivo. Both PL-TLs and LLC-TLs significantly induced *Arg1* mRNA expression in WT and *Tlr4^–/–^* PMNs, but not in *Tlr2^–/–^* or *Myd88^–/–^* PMNs (Figure 6, C and D). We also exposed WT, *Tlr2^–/–^*, and *Myd88^–/–^* PMNs to recombinant ANXA2 protein to confirm the dependence of ANXA2 signaling on TLR2 and MYD88 (Figure 6E). Lastly, we utilized these neutrophils to further clarify the roles of TLR2 and TLR4 ligands as well as other molecules previously reported to induce *Arg1* mRNA expression from neutrophils. Consistent with the above results, the prototypical TLR2 ligand, lipotechoic acid (LTA), was able to induce *Arg1* mRNA expression in WT neutrophils but not in *Tlr2^–/–^* or *Myd88^–/–^* neutrophils. In contrast, the prototypical TLR4 ligand, lipopolysaccharide (LPS), was unable to induce ARG1 in any neutrophil type. We also tested whether 2 cytokines previously reported to induce ARG1 in myeloid cells, IL-10 and IL-4, were in fact capable of doing so in neutrophils. Our results confirm that IL-4 induces *Arg1* mRNA expression in PMNs that is independent of the TLR2/MYD88 axis, consistent with reports that neutrophils express the IL-4 receptor (43). However, IL-10 was incapable of inducing *Arg1* mRNA expression in neutrophils, highlighting the fact that all reported ARG1-inducing ligands should be reconsidered if they were solely tested on murine monocytes and macrophages. Thus, the TLR2/MYD88 pathway mediates ANXA2- and LTA-induced *Arg1* mRNA expression in HDNs, whereas TLR4 and TLR4 ligands do not play a role in this process.

### ANXA2 induces ARG1 gene expression in human peripheral blood neutrophils.

**Figure 7:**
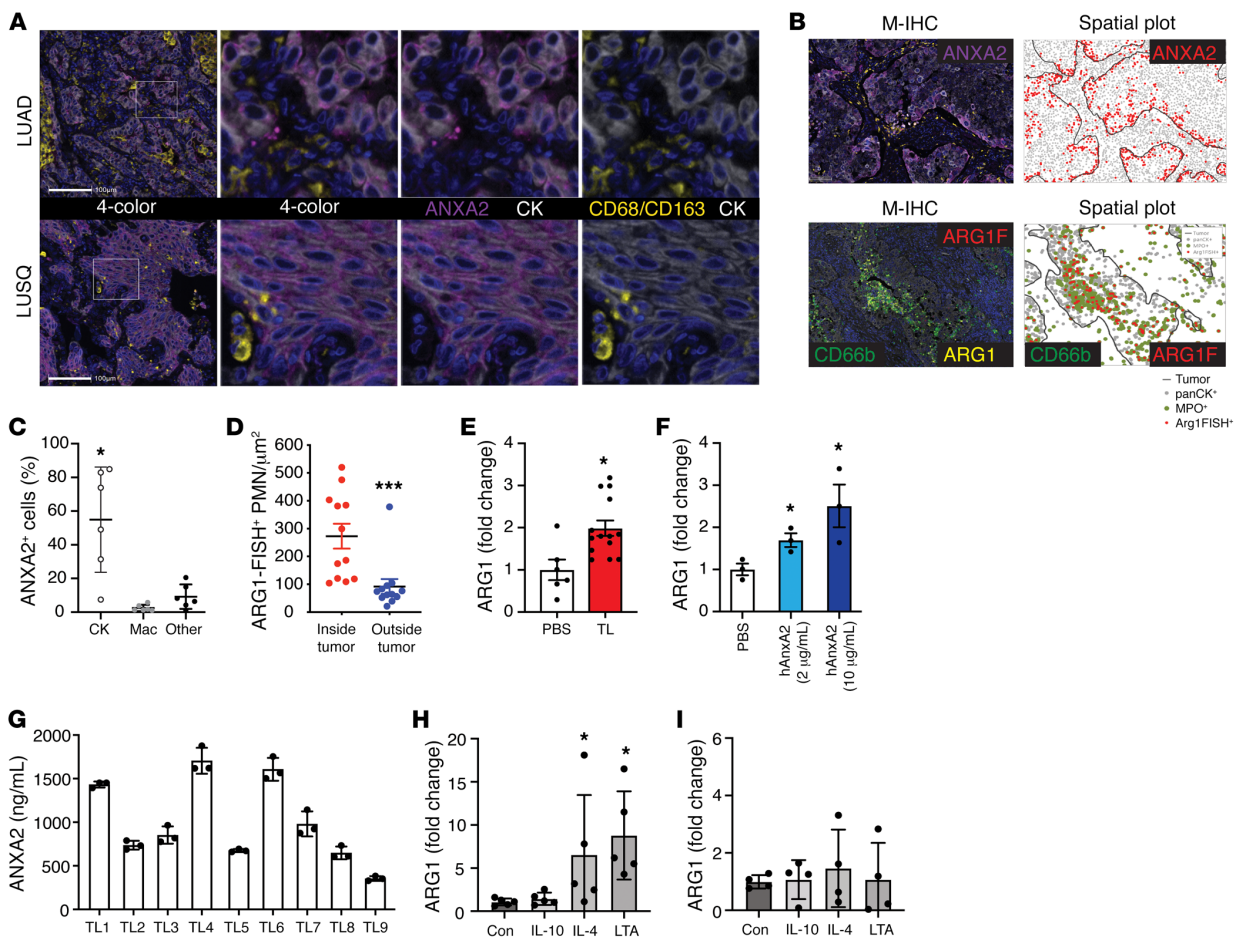
ANXA2 induces *ARG1* gene expression in human neutrophils. (**A**) Representative images from FFPE NSCLC slides (*n* = 6) stained for CD68/CD163 (yellow), ANXA2 (purple), and CK (white). Images farthest to the left are ×20 original magnification, all others are ×40. (**B**) Representative M-IHC and spatial plot images for ANXA2 (purple) staining and CD66b (green), ARG1 IHC (yellow), and ARG1-FISH (red) staining. (**C**) Percentage of ANXA2^+^ cells also staining positively for CK, CD68/CD163, or neither (other). Bars denote ± SEM. (**D**) Tabulation of ARG1-FISH^+^ cells as a function of location either inside or outside the malignant tumor boundary. *n* = 12. Bars denote ± SEM. HDNs from healthy donors were isolated from peripheral blood within 1 hour after blood draw. HDNs were incubated with (**E**) human NSCLC tumor lysate (100 μg/mL) or (**F**) recombinant human ANXA2 for 1 hour. Results expressed as fold change of *ARG1* mRNA expression in the treatment group compared with PBS control. PBS, *n* = 6; tumor lysate, *n* = 14. ANXA2 experiments from a representative experiment in triplicate. (**G**) ANXA2 ELISA from human NSCLC tumor lysates (*n* = 9). Results expressed as ANXA2 protein concentration in ng/mL. (**H**) HDNs from healthy donors as in **E** incubated with IL-10, IL-4, or LTA for 1 hour. Results expressed as fold change in *ARG1* gene expression. *n* = 4 per group. *P < 0.05; ***P < 0.001 by 1-way ANOVA with Tukey’s post hoc test (**C** and **H**) or 2-tailed Student’s *t* test (**D**–**F**). (**I**) Monocyte-derived macrophages were generated from human peripheral blood and incubated with IL-10 (10 U/mL), IL-4 (10 U/mL), or LTA (20 μg/mL) for 1 hour. Results expressed as fold change in *ARG1* gene expression. *n* = 4 per group. *P* > 0.05 by 1-way ANOVA with Tukey’s post hoc test.

To demonstrate the relevance of the above findings to human disease, we performed ANXA2 staining on human LUAD and LUSQ specimens. The M-IHC panel included CD68/CD163 (macrophage cocktail) and CK to determine whether ANXA2 staining was predominantly localized to tumor cells or macrophages. Similar to the findings in the mouse models, ANXA2 was highly expressed in the cancer cell compartment, while macrophage staining for ANXA2 was negligible (Figure 7, A–C). Spatial plot analysis revealed that ANXA2 protein content was predominantly located at the edge of malignant tumor compartment, adjacent to the tumor stroma (Figure 7B). Reanalysis of the ARG1-FISH data and spatial plots showed that ARG1-FISH^+^ cells were also preferentially located within the tumor compartment as opposed to outside of it (Figure 7D), placing ANXA2 protein and *ARG1* transcript in a similar anatomical location.

We tested human NSCLC lysates and recombinant human ANXA2 protein for their ability to induce *ARG1* mRNA expression in human neutrophils, as is the case in mice. Neutrophils were isolated from the high-density fraction of peripheral blood from healthy volunteer donors and cultured with either human NSCLC lysates (100 μg/mL) or human recombinant ANXA2 (2 μg/mL) for 1 hour. Both NSCLC tumor lysates (Figure 7E) and ANXA2 protein (Figure 7F) induced *ARG1* mRNA expression by approximately 2-fold in naive HDNs. ANXA2 ELISA was performed on NSCLC resection specimens (*n* = 9), demonstrating that human lung cancers possess a high concentration of ANXA2 protein and lending physiological relevance to the stimulation assays (Figure 7G). Lastly, we exposed human peripheral blood neutrophils to LTA and IL-4 stimulation to confirm their ability to stimulate *ARG1* mRNA expression, as shown above for mouse neutrophils (Figure 7H). Notably, human monocyte-derived macrophages (MDMs) did not produce *ARG1* transcripts in response to IL-4 or LTA (Figure 7I), despite harboring the receptors for both (43–46). These results suggest that the inability of macrophages to express *ARG1* transcripts is unlikely to be related to TLR2 or IL-4 signaling and more likely related to regulation of the *ARG1* gene in macrophages.

## Discussion

In this report, we provide 3 important contributions to our understanding of the role of ARG1 in cancer. First, we found that ARG1 protein is mainly limited to cells of the neutrophil lineage within the lung TME and found no evidence that monocytes or macrophages could produce and release this immunosuppressive substance. Furthermore, most TANs stained positively for ARG1 and not just the “suppressive” PMN-MDSC population. Second, using a panel combining ARG1 IHC and ARG1-FISH, we discovered that TANs actively produce *ARG1* transcripts in human NSCLC. This result highlights the plasticity of neutrophil responses to cancer. Traditional thinking would support the concept that neutrophils undergo apoptosis or NETosis shortly after exocytosing or dumping their granular contents within the TME. Our results show that TANs are capable of actively transcribing ARG1. These results are consistent with other recent studies that point to longer half-lives of neutrophils in cancer than previously described (47). Third, we have identified the mechanism by which TANs generate ARG1, which involves what we believe is a novel ANXA2/TLR2/MYD88 axis. ANXA2 is involved in multiple cellular processes, including extracellular matrix regulation and cell-cell interactions (48). In the context of cancer, aberrant expression of ANXA2 has been reported in multiple malignancies and demonstrated to predict poor patient outcomes specifically in NSCLC (49, 50). Since ANXA2 can be secreted from the cell (51), we suspect that it is acting in a paracrine fashion within the TME, where its concentration would be highest. We pursued ANXA2 as the likely source of ARG1 induction based on the results of our screen and on prior reports that ANXA2 is a ligand for TLR2 and TLR4 (39). Since TLR2 and TLR4 are inflammatory sensors highly expressed on TANs (and specifically ARG1^+^ TANs), we hypothesized that the TME-derived ANXA2 induced ARG1 in PMNs through either TLR2 or TLR4 signaling. Indeed, an ANXA2-blocking peptide eliminated the ability of TLs to induce *Arg1* mRNA expression in neutrophils. Similarly, recombinant ANXA2 protein was able to induce ARG1 gene expression. Using neutrophils isolated from *Tlr2^–/–^*, *Tlr4^–/–^*, and *Myd88^–/–^* mice, we were able to show that ANXA2 signals through the TLR2/MYD88 pathway to induce *ARG1* gene expression. Lastly, we were able to demonstrate that this axis is relevant to human disease by reproducing the key findings using human peripheral blood neutrophils, NSCLC tumor lysates, and recombinant human ANXA2 protein.

The results presented here will impact the field of immunology and cancer immunotherapy in several ways. First, active efforts to therapeutically target monocytes and macrophages, such as through CSF1R or CCR2, may prove beneficial but would not address ARG1-mediated immune suppression. Second, although the active clinical trials employing an ARG1 antagonist may prove successful, targeting the axis driving ARG1 production in neutrophils may prove superior. Obviously, additional studies will be required to justify such a strategy. Similarly, inhibition of TLR2 on neutrophils, in the right context, may accomplish greater inhibition of immunosuppressive activities than ARG1 antagonism alone. Lastly, debate persists as to whether targeting the neutrophil population as a whole (e.g., CXCR2 antagonist) will be required to block the function of these cells within the TME or if just targeting the PMN-MDSC subset would prove equally efficacious and perhaps safer. The results here suggest that most TANs have the capacity to mediate the immunosuppressive activities of ARG1 such that targeting only PMN-MDSCs would not sufficiently inhibit the immunosuppressive burden generated by the neutrophil lineage as a whole.

Several ligands have been previously reported to induce *ARG1* mRNA transcript expression in myeloid lineage cells. We performed a series of experiments testing some of these ligands to place our results in context of prior work. The results suggest that there are several ligands that can induce ARG1 in neutrophils, including LTA, IL-4, and ANXA2. The operative ligand in any instance is likely to be related to the environment in question, especially as it pertains to TLR2 ligands. The results also show that some ligands previously reported to induce ARG1, such as IL-10, stem from experiments performed in mouse monocytes and macrophages and are unlikely to be relevant to human disease. Lastly, it is intriguing that both IL-4 and LTA can induce *ARG1* mRNA transcript expression in human neutrophils but not in human MDMs despite both cell types possessing the receptors for each ligand (43–46). This suggests *ARG1* gene regulation is more likely account for the inability of human macrophages to express ARG1 than IL-4 or TLR2 signaling. This is an area of active investigation in our laboratory.

There are a few potential limitations to our study worthy of discussion. As mentioned in the Introduction, human neutrophils predominantly transcribe *ARG1* while in the BM and store the protein in granules for later use. In contrast, mouse neutrophils induce *ARG1* gene expression upon external stimuli for more immediate use (ARG1 is not stored in neutrophil granules). Despite this fundamental difference, we chose to use mouse models to complement the human-based studies for the following reasons: (a) human TANs produce *ARG1* transcripts within the lung TME, as do murine TANs (the event that we are attempting to study in detail); (b) availability of molecular reagents and genetically engineered models (e.g., *Tlr2^–/–^* mice) to identify the operative mechanisms; and (c) murine PMNs display a high dynamic range for *Arg1* gene expression such that performing assays in these cells allows one to track ARG1 induction using qPCR, which would not be feasible using human PMNs. This last feature is ultimately what enabled us to identify the operative mechanism here. TLs induced *ARG1* mRNA expression in both human and mouse neutrophils, though the overly robust induction in mouse neutrophils is what enabled us to screen molecular weight–based fractions from TLs to identify the fractions harboring activity. The fractions are diluted during the FPLC process, which makes it difficult to identify activity when the signal is in the 2-fold to 3-fold range. Furthermore, most pathway inhibitors (e.g., TLR2) require prolonged incubation times, which is not compatible with ex vivo neutrophil experimentation. Therefore, the availability of the genetically engineered mouse reagents proved essential in determining the operative mechanisms. Ultimately, our strategy here was to uncover the mechanistic process in detail in mouse model systems and validate the key findings in human model systems. Accordingly, human NSCLC TLs induced *ARG1* mRNA expression in human peripheral blood neutrophils, as did recombinant human ANXA2 protein. An additional concern was with respect to our inability to definitively account for the cellular source of all ARG1^+^ and ANXA2^+^ cells in the M-IHC studies and the inconsistencies in neutrophil marker staining. We used CD66b here because it robustly stains neutrophils. However, not all neutrophils stain positively for CD66b and not all CD66b^+^ cells are neutrophils, as eosinophils can also stain for this marker (52). We suspect that all ARG1^+^ cells represent neutrophil lineage cells that did not stain for CD66b and/or MPO as result of either the neutrophils being in a NETotic or necrotic state or a lack of uniform staining of typical neutrophil markers. Similarly, our limited studies suggest that ANXA2 is largely elaborated from the tumor cell compartment, though minor contributions from other cellular sources remain a possibility. More detailed IHC panels (and other controlled experiments) would be required to reach such conclusions, which were deemed beyond the scope here.

In summary, TANs actively transcribe *ARG1* within the TME via what we believe to be a novel ANXA2/TLR2/MYD88 axis. In addition to this finding, our work provides much needed clarification on the cellular sources of ARG1. This study clearly illustrates that ARG1 in human NSCLC is mainly restricted to cells of the neutrophil lineage while excluding a role for ARG1 in the mediation of monocyte and macrophage immunosuppression. Cumulatively, these results demonstrate the origin and source of ARG1 activity within the TME and provide critical mechanistic insight with which to formulate more comprehensive therapeutic targeting strategies to overcome myeloid cell–derived immunosuppression.

## Methods

### Mice.

B6.129S4-*Pten*^tm1Hwu^/J (*Pten^fl/fl^*) and *Stk11*^tm1.1Sjm^/J (*Lkb1^fl/fl^*) mice on pure C57BL/6 backgrounds were acquired from The Jackson Laboratory. *Pten^fl/fl^*; *Lkb1^fl/fl^* (PL) mice were generated by simple crossbreeding. PL mice received an intratracheal dose of 5 × 10^7^ PFU of adenoviral Cre recombinase (AdCre; University of Iowa Viral Vector Core, Iowa City, Iowa, USA) to initiate tumorigenesis between 8 and 10 weeks of age, as previously described (53). PL mice were studied 28 to 30 weeks following AdCre administration and after MRI confirmation of lung tumor.

C57BL/6 WT mice, C57BL.6-*Arg1^tm1Pmu^*/J (*Arg1^fl/fl^*) mice, B6.129P2(SJL)-*Myd88tm1.1Defr*/J (*Myd88^–/–^*) mice, B6.129-*Tlr2tm1Kir*/J (*Tlr2^–/–^*) mice, and B6.B10ScN-*Tlr4lps-del*/JthJ (*Tlr4^–/–^*) mice were all obtained from The Jackson Laboratory. *Ly6G-Cre* mice were provided by Matthias Gunzer (University Duisburg-Essen, Essen, Germany) (54). The *Ly6G-Cre; Arg1^fl/fl^* mice were generated by simple crossbreeding. An assessment of neutrophil function in *Ly6G-Cre; Arg1^fl/fl^* mice is provided in Supplemental Figure 3. Subcutaneous flank tumors were generated by inoculating 0.2 × 10^6^ LLC cells into the flanks of the mice. Tumors were harvested 21 days after inoculation for further analysis.

### Reagents, media, and cell lines.

LLC cells were obtained from ATCC and were cultured in complete DMEM with 10% FBS and penicillin/streptomycin. Splenic T cells were isolated from C57BL/6 mice and were stimulated in T cell medium (RPMI 1640, penicillin/streptomycin, 200 mM L-glutamine, 50 mM β-mercaptoethanol, 1 M HEPES) with or without L-arginine (75 μM) and nor-NOHA (Sigma-Aldrich). Purified anti–mouse CD3 and purified anti–mouse CD28 antibodies were purchased from BioLegend (catalog numbers 100339 and 102122, respectively). IFN-γ, ANXA2, and ARG1 ELISAs were performed using R&D Systems Quantikine ELISA kits. Recombinant ANXA2 (R&D Systems), IL-4, and IL-10 (Life Technologies), LTA (US Biological), and LPS (Sigma-Aldrich) were used at the indicated concentrations.

### Tissue processing.

Single-cell suspensions were generated from saline-perfused mouse lungs using mechanical disruption, followed by a 30-minute digestion at 37°C in RPMI 1640 containing 80 U/mL DNase and 300 U/mL collagenase type 1 (both from Worthington Biochemical). Digested lungs were strained through a 70-μm nylon mesh, centrifuged, lysed (RBCs), washed, strained through a 40-μm mesh, centrifuged, and resuspended in Dulbecco’s PBS (DPBS) with 2% FCS. Cellular viability was determined using trypan blue staining and a TC20 Automated Cell Counter (Bio-Rad).

### Flow cytometry.

Single-cell suspensions were incubated with mouse TruStain FcX (BioLegend) for at least 15 minutes on ice prior to a 30-minute immunostaining with fluorochrome-conjugated antibody cocktails (Supplemental Table 2). After the extracellular staining, cell pellets were washed using 2% FBS in PBS and Fixable Viability Dye eFluor 780 (eBioscience) was used following the manufacturer’s instructions to exclude dead cells. Stained cells were then washed and fixed with IC fixation buffer (eBioscience) and stored at 4°C and protected from light until the time of analysis. Samples were analyzed using the BD FACSymphony II cell analyzer. Compensation and gating analysis were done with FlowJo Software (Tree Star). Source and clone information for all antibodies is provided in Supplemental Table 2.

### Neutrophil and monocyte isolation.

Mouse peripheral blood neutrophils were collected according to a published protocol (8). Briefly, whole blood from euthanized mice was collected by cardiac puncture. Blood was diluted with 5 volumes PBS containing 0.5% BSA. Diluted blood was run in a discontinuous Histopaque gradient (1.077 and 1.119 g/mL). HDNs were collected from the 1.077-1.119 g/mL interface, while LDNs were collected from the plasma-1.077 g/mL interface. Red blood cells were lysed using ACK lysis buffer. BM neutrophils, splenic neutrophils, and TANs were purified using the Miltenyi Biotec UltraPure Ly6G selection kit. For functional and gene expression assays, HDNs and LDNs were also isolated via Ly6G column purification. Neutrophils were isolated during the course of the study from the following lines of mice: C57BL/6, PL, *Tlr2^–/–^*, *Tlr4^–/–^*, *Myd88^–/–^*, and *Ly6G-Cre/Arg1^fl/fl^*.

Human peripheral blood was obtained from healthy volunteers for the purpose of isolating neutrophils and generating MDMs on an IRB-approved protocol. For neutrophil isolation, human peripheral blood was collected into EDTA-containing tubes, mixed with an equal volume of 3% Dextran T500 (Sigma-Aldrich) solution, and incubated for 30 minutes at room temperature. The top clear leukocyte-rich fraction was collected and laid slowly on top of Histopaque-1077 (Sigma-Aldrich, 10771) density solution. After a 30-minute density centrifugation at 400*g* and room temperature without brake, cells in the high density (at the bottom) fraction were resuspended in RBC lysis buffer for 3 minutes. Following PBS neutralization, cells were centrifuged and resuspended in wash buffer (PBS, 2% FBS, 1 mM EDTA) for subsequent use. The cells from the high-density fraction were subjected to neutrophil purification using a CD66b positive immunomagnetic selection kit (STEMCELL Technologies). For MDM generation, human peripheral blood mononuclear cells (PBMCs) were collected from fresh normal human blood after Histopaque-1077 density gradient centrifugation. CD14^+^ monocytes were isolated from the fresh PBMCs using a negative immunomagnetic selection kit (STEMCELL Technologies). The MDMs were generated from the purified CD14^+^ monocytes as previously described (55). Briefly, monocytes were incubated at density of 10 × 10^6^ cells per 15-cm^2^ non–TC-treated culture dish in the RPMI 1640 medium supplemented with 10% heat-inactivated FBS, 1 mM sodium pyruvate, nonessential amino acids, 2 mM L-glutamine, 55 μM β-mercaptoethanol, antimycotic antibiotic, and human recombinant M-CSF1 (PeproTech) at a final concentration of 50 ng/mL for 6 days. The differentiation medium was removed, the MDMs were rinsed with DPBS (Mg^2+^/Ca^2+^ free) and treated with detachment solution (DPBS, 2 mM EDTA, 0.5% BSA), lifted by gentle scraping, harvested, counted, and used for experiments.

### Gene expression analysis.

Total RNA was isolated from column-purified PMNs from the BM, spleen, peripheral blood, and tumor using an RNeasy Mini Kit (QIAGEN). cDNA was generated from 2 mg of total RNA using SuperScript II Reverse Transcriptase and oligo(dT) (Life Technologies). The expression of indicated target genes was analyzed using a StepOnePlus Real-Time PCR System and TaqMan primer/probe sets (Applied Biosystems), with all reactions run in triplicate. The delta cycle threshold values (ΔCT) were calculated using *Gapdh* as the endogenous housekeeping gene. Results are represented as fold-change values.

### FPLC.

Whole-tumor lysates from PL and LLC (21 day) tumors were generated using a tissue homogenizer. Protein concentrations were measured using a Pierce BCA Protein Assay Kit according to the manufacturer’s instructions. TL samples (~1 mg/mL) were manually injected into the sample loop of FPLC model AKTA900 (GE Healthcare). The Superdex 200 Increase column (Cytiva) was equilibrated with PBS and samples were eluted with PBS. Samples from tubes 1–23 were collected, with each fraction being 0.7 mL in volume.

### MS.

A volume of each FPLC fraction containing 5 μg total protein was aliquoted to a separate tube and diluted with 100 mM ammonium bicarbonate buffer. An equal volume of 8 M urea was added and the sample was vortexed briefly. Disulfide bond reduction was carried out using dithiothreitol (DTT) at a 10 mM final concentration with incubation at 37°C for 1 hour. The samples were removed from heat and alkylated using chloroacetamide at a final concentration of 55 mM, followed by an additional 30-minute incubation period at room temperature in the dark. The alkylation reaction was quenched with additional DTT (10 mM) and the sample solution was diluted further with ammonium bicarbonate (100 mM) to reduce the urea concentration to 1 M. For digestion, trypsin was added in a 1:20 enzyme/substrate ratio along with CaCl_2_ (1 mM final concentration) and the samples were incubated again at 37°C overnight. Samples were removed from the incubator the following morning and the digestion step was halted by adding 2 μL of neat trifluoroacetic acid (TFA). Each solution was desalted with a Millipore C-18 ZipTip using a protocol based on the manufacturer’s recommendations. The resulting peptides were eluted from the tip in 75%:25% acetonitrile/0.1% TFA solution (volume/volume) and subsequently dried by speed-vacuum.

For LC-MS analysis, the dried peptides were resuspended in 20 μL of a 0.1% formic acid/acetonitrile solution (98%:2% by volume). A 15 μL aliquot was injected onto a 2 cm (o.d.) × 100 μm (i.d.) capillary trap column connected to a 250 mm × 75 μm capillary analytical column in a vented configuration. Both trap and column used Michrom Magic C18 aQ packing material, with the trap containing packing material of 200 Å pore size and the column of 100 Å pore size. The peptide solution was gradient eluted from the analytical column with a Thermo Fisher Scientific Easy-nanoLC II HPLC system and analyzed by a Thermo Fisher Scientific Orbitrap Elite mass spectrometer using an MS1 resolution of 240 k in the Orbitrap and collisional induced dissociation (CID) in the ion trap to generate fragment ions of the top-20 most abundant precursor ions observed between 400 and 1800 *m*/*z*. The dynamic exclusion duration was set at 15 seconds. The elution gradient used was a mixture of solvents “A” consisting of water (with formic acid at 0.1%) and “B” containing acetonitrile (with formic acid at 0.1%) starting at 7% B to 35% B over 60 minutes at 400 nL/min, followed by an increase to 50% B over 10 minutes, and a column wash at 95% B for 3 minutes.

The raw data were searched in the Thermo Fisher Scientific Proteome Discoverer software package (v2.2) with the Sequest HT search engine and the Percolator statistical validation node using both a recent mouse database FASTA file from UniProt (https://www.uniprot.org/proteomes/UP000000589 Accessed June 27, 2018) and a FASTA file from the common Repository of Adventitious Proteins (https://www.thegpm.org/crap/ Accessed January 29, 2015) containing background contaminant proteins. The search precursor and fragment tolerances were set at 10 ppm and 0.6 Da, respectively, and dynamic modifications of +15.995 Da for oxidation at methionine and +57.021 Da for carbamidomethylation at cysteine were used. The resulting list of protein hits was further filtered at the peptide level using a 1% false-discovery rate.

### Immunoblotting.

Protein concentrations were calculated using a BCA Protein Assay Kit (Pierce, 23225) and separated using NuPAGE 4%–12% Bis-Tris gels (Life Technologies) followed by transfer to nitrocellulose membranes. Equal micrograms of protein from each FPLC fraction were loaded in the gel, and nitrocellulose membranes were blocked for 1 hour at room temperature in 5% milk in PBS plus Tween 20. Primary antibodies were incubated either overnight at 4°C followed by secondary antibody or for 1 hour at room temperature. Membranes were incubated in SuperSignal West Pico Chemiluminescent Substrate (Thermo Fisher Scientific) for 5 minutes and then developed using an X-ray film processer. Primary antibody used was rabbit anti-ANXA2 (Cell Signaling Technology, clone D11G2, catalog 8235; 1:3000).

### ARG1 activity assay.

Cell lysates of column-purified neutrophils from BM, spleen, tumor, and peripheral blood were analyzed by ARG1 activity assay using the ARG1 activity assay kit (Abcam, ab180877) according to the manufacturer’s protocol.

### IFN-γ production assay.

Splenic CD8^+^ T cells from healthy WT mice were isolated using the EasySep Mouse CD8^+^ T Cell Isolation Kit (STEMCELL Technologies). CD8^+^ cells were washed with PBS and resuspended in T cell media and stimulated with anti-CD3 and anti-CD28 antibodies (BioLegend). Neutrophils isolated from tumors were cultured with the CD8^+^ cells with or without L-arginine and nor-NOHA as indicated. Supernatants were collected 24 hours after cell culture and 20 μL of the sample was used for ELISA to determine IFN-γ production.

### Human specimens.

Deidentified human NSCLC specimens, formalin-fixed, paraffin-embedded (FFPE) slides, and peripheral blood samples were obtained on approved protocols via NWBioTrust. In total, *n* = 44 FFPE slides were analyzed by M-IHC (*n* = 22 LUAD and *n* = 12 LUSQ) and *n* = 18 were analyzed by combined FISH/M-IHC.

### M-IHC.

M-IHC staining and multispectral imaging were performed using PerkinElmer’s Opal IHC reagents and Vectra 3.0 automated imaging system as described below. FFPE tumor tissue slides sectioned at 4 μm were baked for 1 hour at 60°C, and then dewaxed and stained on a Leica BOND Rx stainer. Leica Bond reagents were used for dewaxing (Dewax Solution), antigen retrieval and antibody stripping (Epitope Retrieval Solution 2), and for rinsing after each step (Bond Wash Solution). A high-stringency wash was performed after the secondary and tertiary applications using high-salt TBST solution (0.05 M Tris, 0.3 M NaCl, and 0.1% Tween 20, pH 7.2–7.6). OPAL Polymer HRP Mouse plus Rabbit (PerkinElmer) was used for all secondary applications.

Antigen retrieval and antibody stripping steps were performed at 100°C, with all other steps at room temperature. Endogenous peroxidase was blocked with 3% H_2_O_2_ for 8 minutes followed by protein blocking with TCT buffer (0.05 M Tris, 0.15 M NaCl, 0.25% casein, 0.1% Tween 20, pH 7.6 ± 0.1) for 30 minutes. The first primary antibody (Position 1) was applied for 60 minutes followed by the secondary antibody application for 10 minutes and the application of the tertiary TSA-amplification reagent (PerkinElmer OPAL fluor) for 10 minutes. The primary and secondary antibodies were stripped with retrieval solution for 20 minutes before repeating the process with the second primary antibody (Position 2) starting with a new application of 3% H_2_O_2_. The process was repeated until all 6 positions were completed; there was no stripping step after the sixth position. Slides were removed from the stainer and stained with Spectral DAPI (PerkinElmer) for 5 minutes, rinsed for 5 minutes, and coverslipped with Prolong Gold Antifade reagent (Invitrogen/Life Technologies). Antibodies utilized are listed in Supplemental Table 1.

Stained slides were cured for 24 hours at room temperature, and then multispectral images of representative tumor tissue areas from each slide were acquired on either the PerkinElmer Vectra 3.0 Automated Imaging System for 6-plex or Aperio FL (Leica) for 3-plex images. Vectra images were spectrally unmixed using PerkinElmer InForm software and exported as multiple-image TIFFs for analysis in HALO software (Indica Labs). Aperio FL images were imported directly into the HALO software for analysis.

### FISH/M-IHC.

Combined RNA FISH and 4-plex IHC staining assays were carried out using the Leica Bond Rx Autostainer platform. The human *ARG1* FISH assay was performed using an RNA-scope probe, Hs-ARG1 (ACD Bio, 401588). Briefly, FFPE lung tumor tissue slides were baked and loaded onto the autostainer. The slides were dewaxed using Lecia Bond reagents for dewaxing. Antigen retrieval was performed at 95°C for 15 minutes using Leica Epitope Retrieval Solution 2 followed by permeabilization pretreatment with 1% saponin in EGTA-PBS solution at 40°C for 60 minutes. Next, the slides were blocked with H_2_O_2_ for 10 minutes and then incubated with the target RNA probe at 42°C for 120 minutes. After the probe hybridization, the amplification was performed with RNA-scope 2.5 LS Reagent Kit-Brown (ACD Bio, 322100) and detection by Opal fluor 570 (PerkinElmer, FP1488001KT) at 1:500 dilution for 10 minutes. The RNA FISH assay with positive control probe Human PPIB (ACD Bio, 313908) and negative control probe Dapb (ACD Bio, 312038) was also performed in the adjacent tissue sections for assay quality controls. After completion of the *ARG1* FISH, the stripping was done at 100°C for 20 miutesn using Leica Epitope Retrieval Solution 2 and immediately followed by M-IHC staining against 4 cellular protein markers: CD66b, ARG1, MPO, and panCK. The M-IHC staining step cycles were the same as described above for M-IHC. The staining conditions for each antibody and the RNA probe are listed in Supplemental Table 1.

### Image analysis.

Cellular images were analyzed with HALO image analysis software. After cells were detected based on nuclear recognition (DAPI stain), the fluorescence intensity of the cytoplasmic areas of each cell was measured. A mean intensity threshold above background was used to determine positivity for each fluorochrome within the cytoplasm, thereby defining cells as either positive or negative for each marker. The positive cell data were then used to define colocalized populations and to perform spatial analysis. Final HALO analysis results were exported into Excel (Microsoft), where data tabulation was performed. For ARG1-FISH/M-IHC multiplex images, analysis was performed using the HALO Software v3.1 FISH-IF module (Indica Labs). ARG1-FISH^+^ cells were counted where the cellular marker colocalization with one of more FISH spots was detected.

### Statistics.

Data are expressed as the mean ± SEM, unless otherwise indicated. Statistical analyses were performed using Prism 8 (GraphPad). Multiple comparisons were assessed using 1-way ANOVA with Tukey’s post hoc test. Direct comparisons were assessed using a 2-tailed Student’s *t* test. *P* values of less than 0.05 were considered statistically significant.

### Study approval.

All animal experiments used age- and sex-matched mice and were conducted at the Fred Hutchison Cancer Research Center using protocols approved by the Institutional Animal Care and Use Committee. All human studies were reviewed and approved by the Fred Hutchinson Cancer Research Center IRB, Seattle, Washington. Human NSCLC tumor lysates were obtained from the Fred Hutch Lung SPORE biospecimen resource on an IRB-approved protocol.

## Author contributions

HZ designed the project, performed experiments, interpreted data, crafted figures, and wrote the manuscript. XZ performed and analyzed all M-IHC studies. JK assisted in the design and performance of experiments. TJF and JWK assisted with experiments, design, and data interpretation. TP, MAV, and SM performed experiments. TWR provided statistical support. AMH assisted in study design, data interpretation, crafting of the figures, and writing the manuscript.

## Supplementary Material

Supplemental data

## Figures and Tables

**Table 1:**
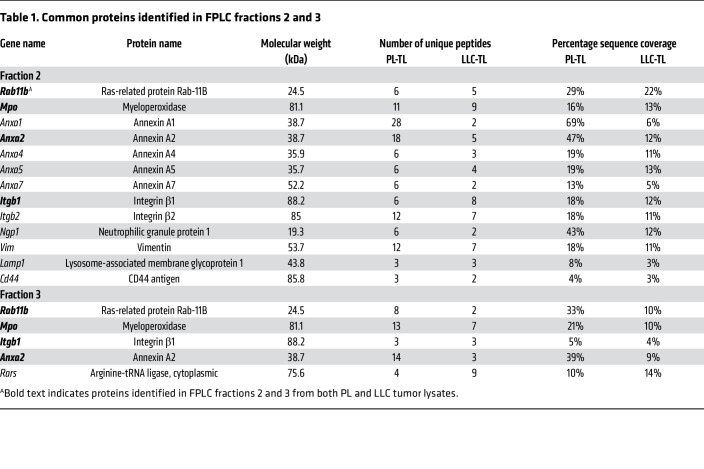
Common proteins identified in FPLC fractions 2 and 3

## Version 1 11/15/2022

Electronic publication
