# Supplementary material for: Annexin A2/TLR2/MYD88 pathway induces arginase 1 expression in tumor-associated neutrophils
Source: J Clin Invest. 2022 Nov 15;132(22):e153643. doi: 10.1172/JCI153643 (PMC9663166; doi:10.1172/JCI153643)
Supplement: Supplemental data [file jci-132-153643-s065.pdf]

Supplementary Materials

Supplementary Table 1. M-IHC panel antibodies for human NSCLC

Supplementary Table 2. Flow antibodies utilized for mouse experiments

Supplementary Table 3. Cytokine array analyte list

Supplementary Figure 1. Multiplexed-immunohistochemistry panel validation

Supplementary Figure 2. Fluorescent in situ hybridization (FISH) panel validation

Supplementary Figure 3. Arg1-deficient neutrophils display normal function

Supplementary Figure 4. Cytokine array from PL mice

Supplementary Table 1

Panel 1. Arginase-1 6-plex for human					
**Position**	**Antibody**	**Clone / Host**	**Company / Item**	**Concentration**	**OPAL Fluor**
1	ARG1	D4E3M/Rabbit	Cell Signaling/93668	1:400 (0.25ug/ml)	Opal 520
2	CD66b	G10F5/mouse	BD Pharm./555723	1:250 (2ug/ml)	Opal 570
3	CD14	EPR3653/Rabbit	Cell Marque/114R-14	1:150 (0.125ug/ml)	Opal 620
4	CD68	PG-M1/mouse	Dako/M0876	1:800 (0.05ug/ml)	Opal 540
5	CD163	EP324/Rabbit	Bio SB/BSB3276	1:1600(0.06ug/ml)	Opal 650
6	CK-ae1/3	AE1/AE3/mouse	Dako/M3515	1:500 (0.33ug/ml)	Opal 690

Panel 2. Arginase-1 FISH and MPO 6-plex for human					
**Position**	**Antibody**	**Clone / Host**	**Company / Item**	**Concentration**	**OPAL Fluor**
1	ARG1 RNA probe	Hs-Arg1	ACD Bio/401588	Neat (ready to use)	Opal 570
2	CD66b	G10F5/mouse	BD Pharm./555723	1:250 (2ug/ml)	Opal 520
3	ARG1 IHC	D4E3M/Rabbit	Cell Signaling/93668	1:400 (0.25ug/ml)	Opal 620
4	MPO	E1E71/Rabbit	Cell Signaling/14569	1:800 (0.125ug/ml)	Opal 540
5	CK-ae1/3	AE1/AE3/mouse	Dako/M3515	1:500 (0.33ug/ml)	Opal 690

Panel 3. Annexin A2 3-plex for human					
**Position**	**Antibody**	**Clone / Host**	**Company / Item**	**Concentration**	**OPAL Fluor**
1	Anxa2	D11G2/Rabbit	Cell Signaling/8235	1:2000 (0.05ug/ml)	Opal 520
2	CD68/ CD163	PG-M1/mouse<br>EP324/Rabbit	Dako/M0876<br>Bio SB/BSB3276	1:800 (0.05ug/ml)<br>1:1600(0.06ug/ml)	Opal 570
3	CK-ae1/3	AE1/AE3/mouse	Dako/M3515	1:500 (0.33ug/ml)	Opal 690

Panel 4. Annexin A2 3-plex for mouse					
**Position**	**Antibody**	**Clone / Host**	**Company / Item**	**Concentration**	**OPAL Fluor**
1	Anxa2	D11G2/Rabbit	Cell Signaling/8235	1:2000 (0.05ug/ml)	Opal 520
2	F4/80	D2S9R/Rabbit	Cell Signaling/70076	1:4000 (0.25ug/ml)	Opal 570
3	CK-wss	WSS/Rabbit	Dako	1:20,000 (0.535ug/ml)	Opal 690

Supplementary Table 2

Flow antibodies utilized for mouse experiments				
**Antibody**	**Clone / Host**	**Company / Item**	**Concentration**	**Fluorochrome**
CD45	30-F11/Rat	Biolegend / 103128	1:800 (0.000625mg/mL)	Alexa Fluor®700
CD11b	M1/70 /Rat	BD Biosciences/ 563168	1:400 (0.0005mg/mL)	BV711
CD11b	M1/70 /Rat	BD Biosciences/ 561098	1:133 (0.0015mg/mL)	PE-Cy™7
Ly6C	HK1.4/Rat	Biolegend/ 128041	1:400(0.0005mg/mL)	BV785
Ly6G	1A8/Rat	BD Biosciences/ 565964	1:200(0.001mg/mL)	BUV395
Ly6G	1A8/Rat	BD Biosciences/ 562700	1:800 (0.00025mg/mL)	PE-CF594
TLR2	QA16A01/Mouse	Biolegend/153006	1:40(0.01mg/mL)	APC
TLR4	SA15-21/Rat	Biolegend/ 145404	1:160(0.00125mg/mL)	PE
Arg1	Arg1/Sheep	R&D System/ IC5868F	1:20(10uL/test)	FITC
Fixable Viability Dye	NA	eBioscience /65-0865-18	1:1000	eFluor™ 780
Fc Block	Fcγ R III/II, Ly-17/Rat	Biolegend/ 101320	1:133(0.003mg/mL)	NA

Supplementary Table 3

Cytokine Array

Coordinate	Analyte/Control	Entrez ID	Alternate Nomenclature
A3,4	Adiponectin/Acrp30	11450	AdipoQ
B15, 16	CCL21/6Ckine	18829	exodus-2, SCYA21, SLC, TCA-4
C9,10	Coagulation Factor III/Tissue Factor	14066	TF, CD142, Thromboplastin
E17, 18	ICAM-1/CD54	15894	
F9, 10	IL-1β/IL-1F2	16176	
F17, 18	IL-4	16189	B cell-stimulatory factor-1
G1, 2	IL-10	16153	CSIF
G7, 8	IL-13	16163	
H5, 6	Lipocalin-2/NGAL	16819	Siderocalin, 24p3
H7, 8	LIX	20311	CXCL5, GCP-2, ENA-78
H15, 16	MMP-9	17395	Clg4b, Gelatinase B, GELB
H17, 18	Myeloperoxidase	17523	MPO

Supplementary Figure 1

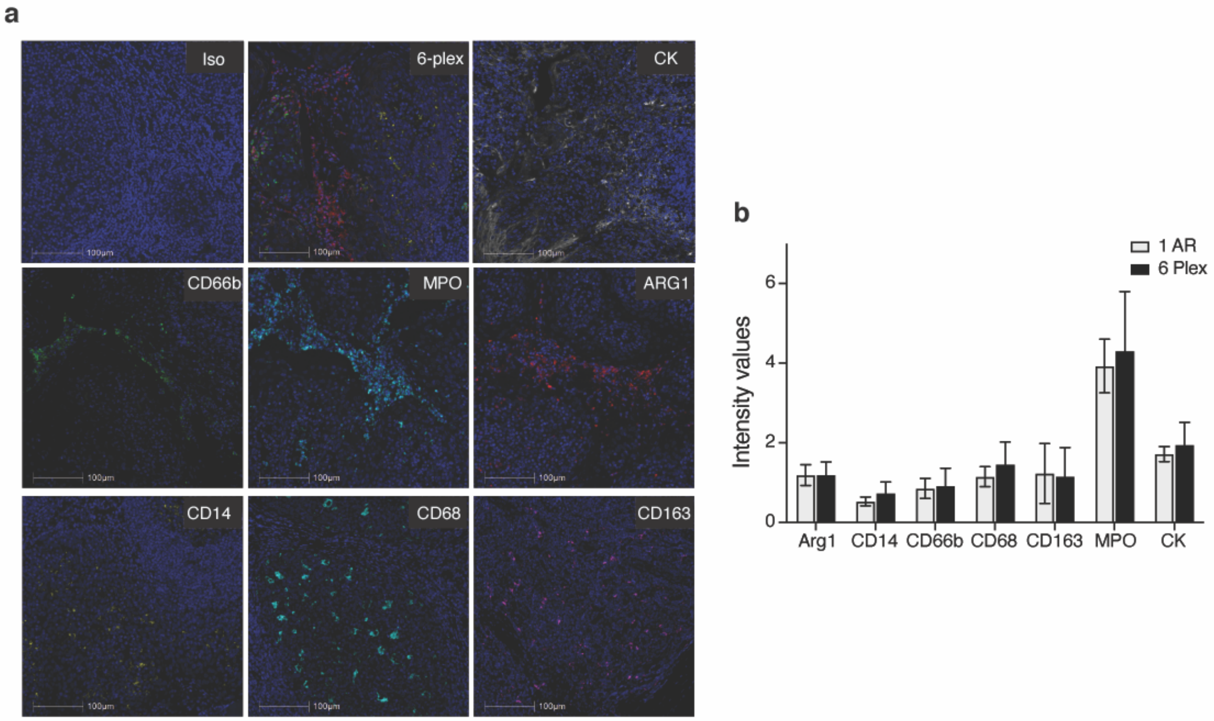

Supplementary Figure 1. **Multiplexed-immunohistochemistry panel validation**. Validation of the 6-plex M-IHC panel from Figure 1 (CK, ARG1, CD66b, CD14, CD68, CD163) was performed using human tonsil. Validation used one antigen retrieval (1AR) compared to six antigen retrievals stained for each individual marker according to its position on the 6-plex panel (an identical procedure was performed for the panel including ARG1 FISH and MPO). Corresponding isotype control IgG or IgM was stained on the same position as its corresponding antibody through the six staining cycles. The signal strength for each marker signal after being subject to multiple cycles of antigen retrieval and antibody stripping on the 6-plex stained slide was compared to the baseline signal levels on the 1AR slide. The validation staining experiment was run twice per panel, in separate experiments. **A**) Representative images of individual markers stained on tonsil for 1AR or the final position on the 6-plex in addition to isotype control. The markers include: DAPI (blue), CK (white), CD66b (green), MPO (cyan), ARG1 (red), CD14 (yellow), CD68 (cyan), and CD163 (magenta). **B**) Tabulation of marker signal strenth at baseline (1AR) vs. final 6-plex for each marker used. Results expressed as mean value +/- STD (N=15 fields per slide). All P values > 0.05 (Student's t-test).

Supplementary Figure 2

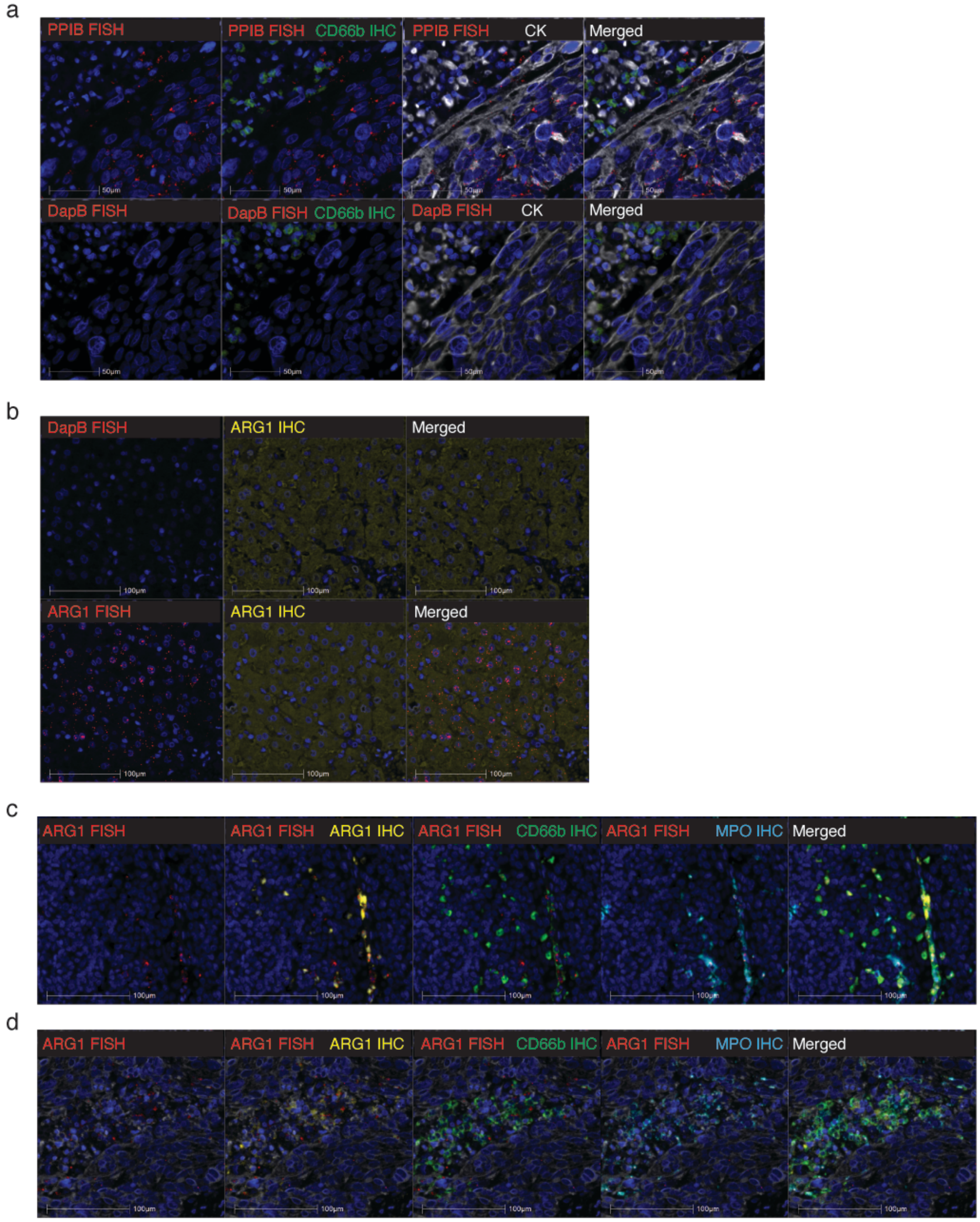

Supplementary Figure 2. Fluorescent in situ hybridization (FISH) panel validation. Arg1 FISH was carried out using the RNAscope assay kit from ACD Biosciences. A) Representative images from Positive (PPIB housekeeping gene) and negative (DapB bacterial gene) controls were performed on lung cancer FFPE tissues with inclusion of CD66b (green) and CK (white) IHC markers for reference. B) Representative images from DapB negative control and Arg1 FISH probe (red) were validated on liver tissue with inclusion of ARG1 IHC marker (yellow) for reference. C) Representative images from ARG1 FISH staining on tonsil FFPE tissue with inclusion of ARG1 (yellow), MPO (cyan), and CD66b (green) IHC markers for reference. D) Representative images from ARG1 FISH probe (red) staining on lung cancer FFPE tissue with inclusion of ARG1 (yellow), CD66b (green), and MPO (cyan) IHC markers for reference. All stained slides were scanned on a Polaris multispectral imaging system (Akoya) and image analysis was performed using the ISH/IF module v3.1 Halo software (Indica Labs).

Supplementary Figure 3

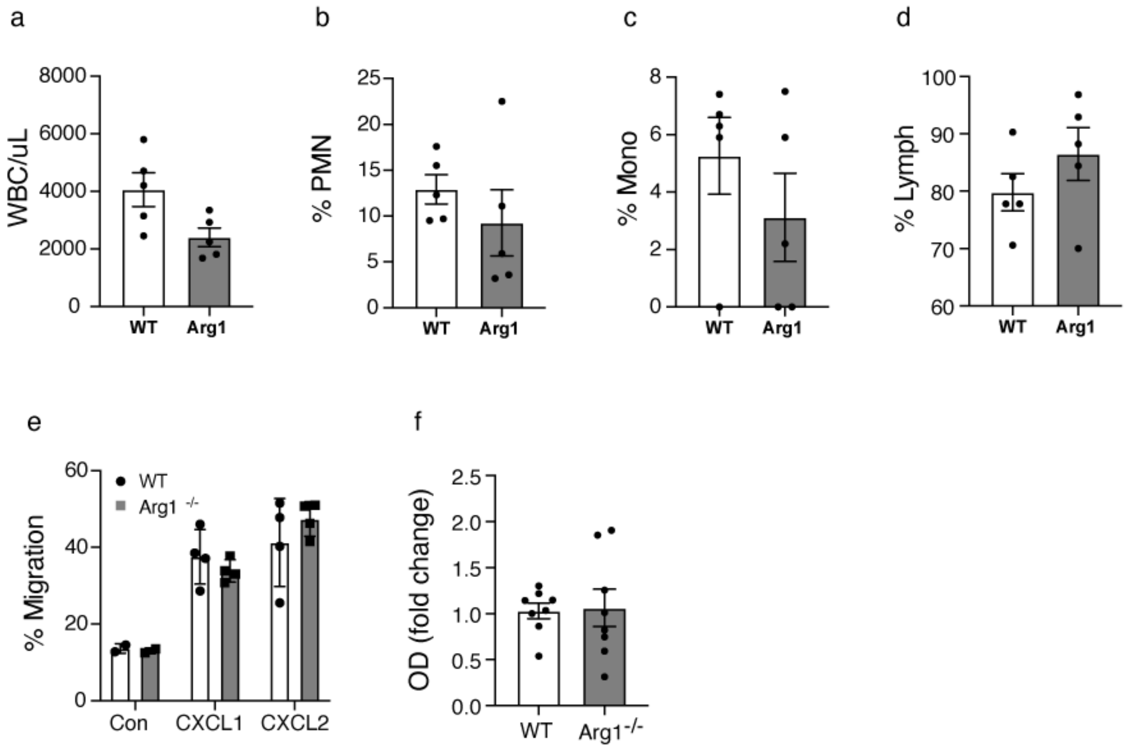

Supplementary Figure 3. Arg1-deficient neutrophils display normal function. Complete blood counts (CBC) were obtained from WT and Ly6G/Arg1$^{fl/fl}$ mice. Results are expressed as A) white blood cell count (WBC) and differential counts (%) for B) neutrophils, C) monocytes, and D) lymphocytes. N=5 mice each group. Bars +/- SEM. P > 0.05 for each comparison (t-test). E) Modified Boyden Chamber chemotaxis assay for WT and Arg1$^{-/-}$ neutrophils utilizing CXCL1 and CXCL2 as the chemoattractants. N=4 each group. Results expressed as % of cells migrated. Bars +/- SEM. P > 0.05 for each condition (ANOVA). F) Phagocytosis assay on WT and Arg1$^{-/-}$ neutrophils utilizing fluorscently labeled E. coli bioparticles. Results expressed as fold change in optical density (OD). Bars +/- SEM. P > 0.05 (t-test).

Supplementary Figure 4

a

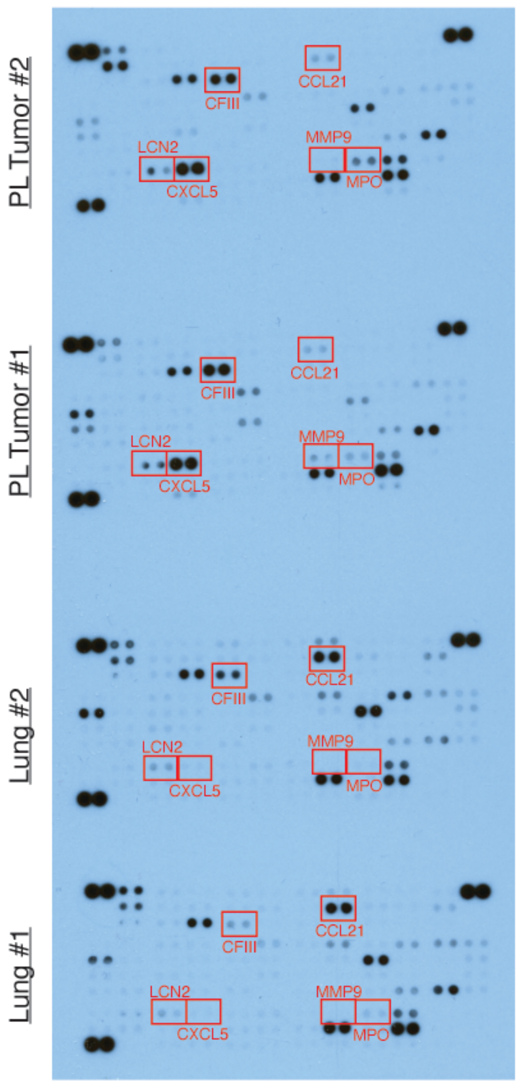

b

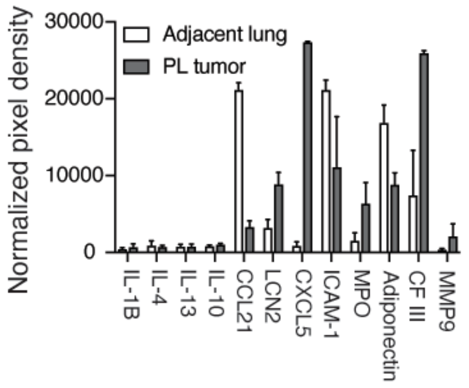

Supplementary Figure 4. Cytokine array. a) Lysates from PL tumors and adjacent lung tissue were subjected to cytokine array. Each specimen was blotted in duplicate and involved N=2. Select cytokines have been highlighted in red boxes. b) The array was scanned and the pixel density for each blot was calculated. Results are shown for select cytokines and presented as normalized pixel density.
